# Pharmacological effects and mechanisms of curcumin in animal models of Parkinson’s disease: a systematic review and meta-analysis

**DOI:** 10.3389/fphar.2026.1779921

**Published:** 2026-03-09

**Authors:** Bowen Pang, Qiang Fu, Huihan He, Xiangyu Guo, Ge Yang

**Affiliations:** College of Traditional Chinese Medicine, Changchun University of Chinese Medicine, Changchun, China

**Keywords:** animal models, curcumin, mechanisms, Parkinson’s disease, systematic review and meta-analysis

## Abstract

**Background:**

Curcumin has been demonstrated to possess promising neuroprotective potential in Parkinson’s disease; however, its overall effects remain inconclusive, and its multiple mechanisms of action have not been systematically summarized.

**Objective:**

This systematic review and meta-analysis aimed to evaluate the pharmacological effects of curcumin in animal models of Parkinson’s disease and to investigate its potential mechanisms involving antioxidant, anti-inflammatory, and neuroprotective effects, thereby providing a theoretical basis for its potential clinical application in Parkinson’s disease.

**Methods:**

A comprehensive search of four databases (EMBASE, PubMed, Web of Science, and the Cochrane Library) up to August 2025 identified 31 eligible studies involving a total of 552 animals. Methodological quality was assessed using the SYRCLE risk of bias tool. Standardized mean differences (SMDs) were calculated to evaluate the effects of curcumin on motor function, neurochemistry, inflammation, and oxidative stress in animal models of Parkinson’s disease.

**Results:**

The results demonstrated that curcumin intervention improved motor function in animal models of Parkinson’s disease, as evidenced by increased locomotor distance in the open field test (SMD = 1.25) and elevated mean velocity (SMD = 1.42), prolonged latency to fall in the rotarod test (SMD = 2.49), shortened descent time in the pole test (SMD = −1.16), and reduced traversal time on the balance beam (SMD = −2.27). Curcumin exhibited neuroprotective effects through increasing the number of tyrosine hydroxylase-positive cells (SMD = 2.12), maintaining dopamine levels (SMD = 4.11), and elevating 3,4-dihydroxyphenylacetic acid concentrations (SMD = 3.15). Regarding anti-inflammatory effects, curcumin significantly reduced multiple inflammatory markers, including interleukin-6 (SMD = −4.73), interleukin-1β (SMD = −3.30), tumor necrosis factor-α (SMD = −3.19), and nitric oxide (SMD = −4.91). With respect to antioxidant activity, curcumin significantly reduced malondialdehyde levels (SMD = −4.69) while increasing the activities of superoxide dismutase (SMD = 3.90), glutathione (SMD = 2.08), and catalase (SMD = 2.00).

**Conclusion:**

Curcumin demonstrates significant neuroprotective effects in Parkinson’s disease animal models, improving motor deficits and neuronal integrity likely through multi-target mechanisms involving anti-inflammatory and antioxidant pathways.

**Systematic Review Register:**

https://www.crd.york.ac.uk/PROSPERO/view/CRD420251131257, identifier CRD420251131257

## 1 Introduction

Parkinson’s disease (PD) is the second most common progressive and irreversible neurodegenerative disorder after Alzheimer’s disease, primarily characterized by motor dysfunction, including tremor, rigidity, bradykinesia, and postural instability (Bloem et al., 2021). As the disease progresses, patients with PD not only experience motor symptoms but may also develop cognitive impairment, mood fluctuations, sleep disturbances, and autonomic nervous system dysregulation, which severely compromise their quality of life and pose substantial challenges to families, society, and healthcare systems (Schapira et al., 2017). According to global statistics from 1996 to 2023, the worldwide mortality from PD increased from 190.7 thousand in 2000 to 427.1 thousand in 2023, representing a growth rate of 123.8% (Collaborators, 2024). Recent estimates from the Global Burden of Disease Study 2021 suggest that approximately 11.77 million people are currently living with Parkinson’s disease worldwide (Luo et al., 2024)A recent modeling study published in the British Medical Journal in March 2025 projected that the global number of PD patients will reach 25.2 million by 2050 (Su et al., 2025).

The pathogenesis of PD remains incompletely elucidated, with the primary pathological hallmarks including the loss of dopaminergic neurons and the formation of Lewy bodies resulting from α-synuclein aggregation (Poewe et al., 2017). Multiple hypotheses have been proposed to explain the underlying mechanisms of PD, including neuroinflammation (Marogianni et al., 2020), oxidative stress (Trist et al., 2019), and mitochondrial dysfunction (Ye et al., 2023), while synaptic dysfunction (O'Keeffe and Sullivan, 2018) and autophagy dysregulation (Lizama and Chu, 2021) also play pivotal roles. Additionally, aberrant neural oscillations in the beta frequency band within basal ganglia circuits have been implicated in motor symptom generation, with recent studies highlighting the bidirectional modulation of these oscillations as a potential therapeutic target (Hu et al., 2025). A growing body of evidence highlights the central role of immune system dysregulation in neurodegenerative processes, bridging peripheral inflammation and central nervous system pathology (Chen et al., 2025). Additionally, genetic factors (Cherian et al., 2023) and environmental toxins (Sun et al., 2007) are closely implicated in disease pathogenesis. Current therapeutic strategies for PD predominantly rely on dopamine replacement therapy or dopamine receptor agonists; however, long-term administration is associated with significant adverse effects (such as dyskinesia and cognitive impairment) (Oertel and Schulz, 2016) and provides only symptomatic relief. For advanced cases, deep brain stimulation (DBS) is often employed, yet its efficacy can be influenced by various factors, including diet, as emerging evidence suggests that purine intake may affect DBS prognosis in PD patients (Chang et al., 2025). This highlights the need for integrative approaches that combine pharmacological, surgical, and lifestyle interventions. Given the complex pathophysiology of PD and the limited understanding of its precise mechanisms, no effective curative treatment has been established to date (Vijiaratnam et al., 2021). In recent years, with advancing research, novel therapeutic development has encompassed multiple directions, including gene therapy, neuroprotection, stem cell therapy, and targeted pharmacological interventions. Nevertheless, these approaches continue to face challenges such as unclear clinical efficacy, difficulties in central nervous system delivery, and limitations of animal models in accurately recapitulating disease mechanisms (Bougea, 2024).

Given the multi-factorial pathology of PD and the limitations of current therapies, there is a pressing need for multi-target, neuroprotective agents. Plant-derived metabolites such as curcumin, with known anti-inflammatory and antioxidant properties, represent a promising avenue for investigation. Curcumin is a polyphenolic plant-derived secondary metabolite derived from the rhizomes of turmeric (Curcuma longa L., Zingiberaceae), a plant indigenous to Southeast and South Asia. It has demonstrated favorable effects in neurodegenerative diseases, cardiovascular disorders, cancer, diabetes, and anti-aging applications (Kotha and Luthria, 2019; Nunes et al., 2024), while possessing anti-inflammatory, antioxidant, antiviral, and immunomodulatory properties (Abd El-Hack et al., 2021). It has been reported that curcumin can modulate the gut microbiota, thereby ameliorating motor symptoms in PD (Cui et al., 2022); furthermore, it attenuates mitochondrial dysfunction by suppressing the NOD-like receptor family pyrin domain-containing 3 (NLRP3) inflammasome in microglia (Xu et al., 2023).

Accumulating evidence suggests that curcumin represents a promising pharmacological intervention for PD, with numerous animal experiments having been conducted. However, due to limited sample sizes and insufficient statistical power in individual studies, the overall neuroprotective effects remains inconclusive. Importantly, it remains unclear whether the available preclinical evidence consistently supports the pharmacological effects of curcumin across different experimental models and outcome measures, and how its reported mechanisms of action can be integrated into a coherent framework. Currently, existing research has predominantly focused on single efficacy endpoints with relatively limited findings and has not comprehensively elucidated the multiple mechanisms of action of curcumin in PD treatment. To our knowledge, this study provides the first quantitative synthesis of preclinical evidence on the pharmacological effects of curcumin in Parkinson’s disease models, with a systematic evaluation of heterogeneity across studies. Therefore, this systematic review and meta-analysis aimed to assess the neuroprotective effects of curcumin in animal models of Parkinson’s disease and to summarize its potential mechanisms of action.

This systematic review and meta-analysis aimed at:

- Evaluate the effects of curcumin on motor function, inflammation, oxidative stress, and neuronal parameters in animal models of PD
- Synthesize the potential mechanisms underlying curcumin treatment for PD
- Investigate factors influencing the neuroprotective effects of curcumin in animal models of PD.

## 2 Methods and materials

This study was prospectively registered in the International Prospective Register of Systematic Reviews (PROSPERO) database (registration number: CRD420251131257) and was conducted in accordance with the Preferred Reporting Items for Systematic Reviews and Meta-Analyses (PRISMA) guidelines (Page et al., 2021).The protocol is accessible at PROSPERO (https://www.crd.york.ac.uk/PROSPERO/view/CRD420251131257).

### 2.1 Literature search strategy

Four electronic databases-EMBASE, PubMed, Web of Science, and the Cochrane Library—were systematically searched up to August 2025. The search terms and their synonyms included “curcumin,” “Parkinson disease,” “paralysis agitans,” and “parkinson dementia complex,” which were combined using Boolean operators “AND” and “OR.” The detailed search strategies were adjusted according to the requirements of each database and are provided in the Supplementary Materials.

The reference lists of all included studies were manually screened to identify additional eligible articles. Two reviewers independently performed the literature screening, and any discrepancies were resolved through discussion and consensus. When essential data was missing, the study authors were contacted for further information. All references included were managed and deduplicated using Zotero software.

### 2.2 Inclusion criteria and outcome measures

Inclusion criteria were as follows:

- Preclinical studies conducted on rats or mice, with no restrictions on strain, species, or sex;
- Studies involving animal models of Parkinson’s disease;
- Curcumin administered as the sole therapeutic agent, compared with a blank control or vehicle-treated group; Outcome Measures: a. Primary outcome measures: dopamine level (DA). b. Secondary outcome measures: Motor function (Open field test: total movement distance and average velocity; Rotarod test: latency to fall; Pole test: time to descend; Beam walking test: time to traverse and number of foot slips); Dopaminergic system and neurotransmitter biomarkers: the number of tyrosine hydroxylase-positive (TH^+^) cells, the levels of homovanillic acid (HVA) and 3,4-dihydroxyphenylacetic acid (DOPAC); Inflammatory indicators:interleukin-6 (IL-6), interleukin-1β (IL-1β), tumor necrosis factor-α (TNF-α), and nitric oxide (NO); Oxidative stress markers: glutathione (GSH), superoxide dismutase (SOD), catalase (CAT), and malondialdehyde (MDA).

Exclusion criteria were as follows:

- Without relevant outcome measures or incomplete data;
- Intervention involved curcumin derivatives or curcumin in combination with other therapies;
- Without a clearly defined control group;
- Full text unavailable.

### 2.3 Data extraction

Data extraction was performed independently by two researchers using a pre-designed data extraction form. The extracted information included: basic details of the included studies (first author and year of publication), characteristics of the experimental animals (species, strain, age, weight, and number), Parkinson’s disease model induction agents, route of administration, curcumin intervention dose, and relevant outcome measures. For data presented in graphical form, Get Data 2.20 software was used for data extraction and conversion. Continuous outcomes were extracted as means and standard deviations (SD); if the data were reported as standard error of the mean (SEM), SD was calculated using the formula SD = SEM × (square root of n) (where n is the sample size). In studies with multiple intervention groups, only data from the Parkinson’s disease control group and the curcumin intervention group were extracted for subsequent analysis.

### 2.4 Quality assessment and risk of bias

Two researchers independently recorded data and assessed the risk of bias in the included studies using the SYRCLE risk of bias tool for animal studies (Hooijmans et al., 2014). This tool consists of 10 items, covering six areas: selection bias, performance bias, detection bias, attrition bias, reporting bias, and other biases. The risk of bias for each study was rated as low, unclear, or high using Review Manager 5.4 software. Any discrepancies were reviewed and resolved by a third researcher.

### 2.5 Statistical analysis

This meta-analysis was performed using Review Manager 5.4 and Stata 18 software for data integration and statistical analysis. All outcome measures were predefined as continuous variables, and effect sizes were combined using the mean difference (MD). When the measurement units differed across studies, the SMD was used. For each outcome, pooled effect estimates were calculated with corresponding 95% confidence intervals (CIs). The I^2^ statistic was employed to assess heterogeneity between studies. I^2^ values of 25%–50% were considered low heterogeneity, 50%–75% moderate heterogeneity, and values greater than 75% high heterogeneity. For outcomes exhibiting substantial heterogeneity, subgroup analyses were performed based on predefined study characteristics (e.g., animal species, disease model, dosage, route of administration, and treatment duration). Sensitivity analyses were conducted using Stata 18 by sequentially excluding individual studies (leave-one-out analysis) to assess the robustness of pooled estimates. Statistical significance for pooled effect estimates was defined as a two-sided P value <0.05. No formal adjustment for multiple comparisons was applied, as subgroup and sensitivity analyses were considered exploratory. Additionally, funnel plots and Egger’s test were used to assess the presence of publication bias, with a P-value of <0.05 considered statistically significant for the presence of publication bias.

## 3 Results

### 3.1 Study selection

**FIGURE 1:**
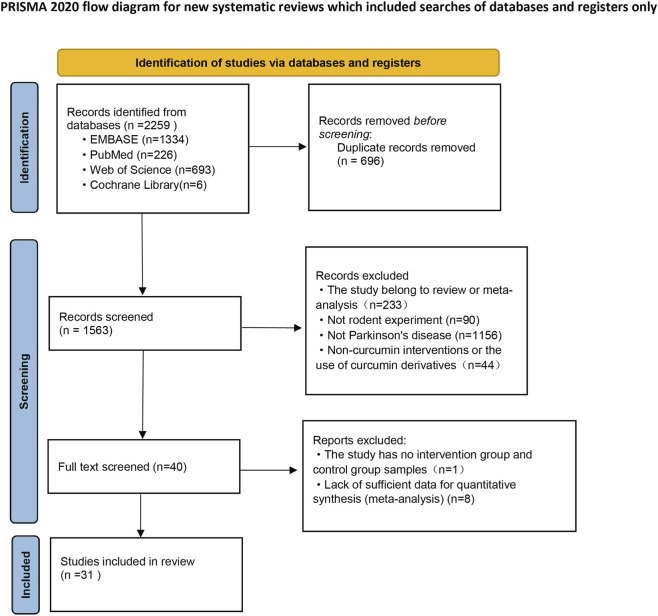
PRISMA flowchart for literature screening.

Our literature search identified a total of 2,259 articles, with 1,334 from EMBASE, 226 from PubMed, 693 from Web of Science, and six from the Cochrane Library. After removing 696 duplicate articles using Zotero software, two reviewers independently screened the remaining 1,563 articles by reviewing their titles and abstracts, resulting in the exclusion of 1,523 articles. Subsequently, 40 full-text articles were assessed, and nine studies were excluded. Among the excluded studies, one did not report the sample sizes for the intervention and control groups, while eight were unrelated to the outcomes of this meta-analysis. In the end, 31 studies were included (Zbarsky et al., 2005; Rajeswari, 2006; Pan et al., 2007; Rajeswari and Sabesan, 2008; Khuwaja et al., 2011; Agrawal et al., 2012; Du et al., 2012; Mansouri et al., 2012; Pan et al., 2012; Lv et al., 2014; He et al., 2015; Cui et al., 2016; Khatri and Juvekar, 2016; Song et al., 2016; Xia et al., 2016; Singh and Kumar, 2017; Sharma and Nehru, 2018; Madiha and Haider, 2019; Bhowmick et al., 2022; Cui et al., 2022; Naser et al., 2022; Zhong et al., 2022; Zhu et al., 2022; Cai et al., 2023; Essawy et al., 2023; Liu et al., 2023; Rathore et al., 2023; Alves et al., 2024; Belviranlı et al., 2025; Cai et al., 2025) (Figure 1).

### 3.2 Study characteristics

Table 1 lists the basic characteristics of the included studies. All studies used male rodents, comprising 304 rats (55%) and 248 mice (45%). Among the 31 studies, the most commonly used species was the Wistar rat (11 studies with a total of 160 rats, 36%), followed by the C57BL/6 mouse (9 studies with a total of 166 mice, 29%). Additionally, SD rats (5 studies with a total of 132 rats, 16%), Swiss albino mice (5 studies with a total of 82 mice, 16%), and Lewis rats (1 study with 12 rats, 3%) were also used. Parkinson’s disease models in the literature included MPTP-induced (10 studies), 6-OHDA-induced (9 studies), rotenone-induced (10 studies), LPS-induced (1 study), and homocysteine -induced (1 study) models. The routes of curcumin administration included gavage (71%) and intraperitoneal injection (26%), with one study not specifying the route of administration. Among the 31 studies, nine used two or three different doses of curcumin in treatment groups and compared them with the same PD control group to investigate dose-response relationships.

**TABLE 1:** Study characteristics of including studies (n = 31).

Study	Species	Strain	Age/weight	PD induction method	n (C/E)	Vehicle/Formulation (purity)	Administration route	Dose (mg/kg)	Treatment duration	Controls (vehicle/Positive)	Related outcome
Lv et al. (2014)	Rats	SD	NR/250–300 g	6-OHDA (300 μg, intracisternal injection, single dose)	10/10	NR	i.p	200	14 days	No treatment, No positive control	SOD, GSH, dopamine level
Sharma and Nehru (2018)	Rats	SD	7∼9weeks/350–400 g	LPS (5 μg, intranigral injection, single dose)	10/10	Vegetable oil	i.p	40	21 days	No treatment, No positive control	TNF-α, IL-1β, NO, GSH
Cai et al. (2023)	Mice	C57BL/6	8weeks/NR	MPTP (20 mg/kg) + probenecid (250 mg/kg, i.p., every 3.5 days for 5 weeks)	6/6	NR	p.o	60	7 weeks	No treatment, No positive control	Open field test, pole test, WB:TH(Snandstriatum)
Cui et al. (2016)	Rats	Lewis	7∼8weeks/260–280 g	Rotenone (1.5 mg/kg, s.c., twice daily for 47 days)	6/6	Gum arabica	p.o	100	50 days	Gum arabica, No positive control	Open field test, GSH, ROS, MDA, WB: TH, AKT, Nrf2
Mansouri et al. (2012)	Rats	Wister	NR/250–300 g	Homocysteine (2 μmol, i.c.v., single dose)	8/8	Ethyl oleate	i.p	50	10 days	Ethyl oleate, No positive control	Open field test
Song et al. (2016)	Rats	SD	8weeks/200 ± 10 g	6-OHDA (20 μg, stereotaxic injection, single dose)	10/30	DMSO + peanut oil (purity: ≥94%)	i.p	5, 10, 20	30 days	Peanut oil +0.05% DMSO, No positive control	SOD, MDA, dopamine level, WB: bFGf, NGF, TrkA, HSP70
Pan et al. (2012)	Mice	C57BL/6	8–10weeks/19–22 g	MPTP (30 mg/kg/day, i.p., daily for 5 days)	6/6	DMSO	i.p	50	5 days	DMSO, No positive control	The cell number of TH, WB:JNK1/2/3, Bax, Bcl-2, COX-4
Rathore et al. (2023)	Mice	Swiss albino	NR/25 ± 5 g	Rotenone (2.5 mg/kg, s.c., once daily for 35 days)	6/6	DMSO + Corn oil	p.o	80	42 days	No treatment, No positive control	Rotarod test, ROS, MDA, CAT, WB: α-Syn, TH, Nrf2, Keap1, LC3-I, LC3-II, Bax, Bcl2, Caspase3, p62
Cai et al. (2025)	Mice	C57BL/6	6∼8weeks/19–23 g	Rotenone (3 mg/kg/day, p.o., daily for 45 days)	6/6	DMSO	p.o	40	45 days	No treatment, No positive control	IL-1β, WB: TH, α-syn, NLRP3, ASC, Caspase-1
Du et al. (2012)	Rats	Wister	50days/200–220 g	6-OHDA (19.8 μg, stereotaxic injection into MFB, single dose)	6/6	Gum Arabic	p.o	200	24 days	Gum arabica, No positive control	DOPAC, HVA, dopamine level, the cell number of TH
Zhu et al. (2022)	Mice	C57BL/6	6∼8weeks/18–22 g	MPTP (30 mg/kg, i.p., daily for 7 days)	12/36	DMSO + Sesame oil	i.p	40, 80, 160	14 days	No treatment, No positive control	Open field test, pole test, the cell number of TH
Madiha and Hider (2019)	Rats	Wister	NR/150–200 g	Rotenone (1.5 mg/kg, s.c., daily for 8 days)	6/6	Sunflower oil	p.o	100	14 days	No treatment, No positive control	DOPAC, dopamine level
Cui et al. (2022)	Mice	C57BL/6	6∼8weeks/20–30 g	MPTP (30 mg/kg/day, i.p., daily for 5 days)	10/10	Saline	p.o	25, 100, 400	28 days	No treatment, No positive control	Open field test, pole test, rotarod test, the cell number of TH, WB: TH, α-syn
He et al. (2015)	Mice	C57BL/6	3weeks/25–30 g	MPTP (40 mg/kg, i.p., single dose)	10/10	Dietary supplementation	p.o	0.5% or 2% w/w in diet	49 days	No treatment, No positive control	The cell number of TH
Zhong et al. (2022)	Mice	C57BL/6	8weeks/20 ± 5 g	MPTP (20 mg/kg, i.p., every 3.5 days for 35 days)	5/5	Gum Arabic solution	p.o	60	49 days	Gum Arabic, No positive control	Open field test, pole test, the cell number of TH, IL-1β, IL-6, TNF-α, WB:TH, SIRT1, NRF2, AIM2, ASC, Caspase-1
Rajeswari and Sabesan. (2008)	Mice	Swiss albino	NR/25–30 g	MPTP (4 × 10 mg/kg at 1-h intervals, i.p., total 40 mg/kg)	8/8	DMSO	i.p	80	7 days	No treatment, No positive control	DOPAC
Agrawal et al. (2012)	Rats	Wister	NR/200–250 g	6-OHDA (10 μg, stereotaxic injection into striatum, single dose)	6/6	0.5% CMC suspension	p.o	60	21 days	No treatment, No positive control	Rotarod test, GSH, CAT, MDA, SOD, DOPAC, HVA, dopamine level
Khatri and Juvekar (2016)	Mice	Swiss albino	NR/22–25 g	Rotenone (1 mg/kg, i.p., once daily for 21 days)	6/18	0.5% CMC suspension (purity: 95%)	p.o	50, 100, 200	21 days	No treatment, No positive control	Rotarod test, MDA, SOD, CAT, GSH
Yang et al. (2014)	Rats	SD	NR/200 ± 10 g	6-OHDA (20 μg, stereotaxic injection into SN/VTA, single dose)	10/30	NR	NR	5, 10, 20	30 days	No treatment, No positive control	Dopamine level, WB:BDNF, TrkB, PI3K, GAPDH
Essawy et al. (2023)	Rats	Wister	12weeks/165–180 g	Rotenone (2.5 mg/kg, p.o., daily for 5 weeks)	10/10	Corn oil	p.o	200	35 days	Corn oil, No positive control	MDA, NO, GSH, SOD, CAT, dopamine level
Singh and Kumar (2017)	Rats	Wister	NR/250–280 g	6-OHDA (8 μg, intranigral injection, single dose)	9/9	CMC solution	p.o	25, 50	21 days	No treatment, No positive control	Rotarod test, beam-crossing task, dopamine level, TNF-α, IL-1β, IL-6, NO, GSH
Zbarsky et al. (2005)	Rats	SD	NR/200 g	6-OHDA (12 μg, stereotaxic injection into MFB, single dose)	6/6	10% Cremophor EL (purity: 98%–99%)	p.o	50	4 days	10% Cremophor, No positive control	DOPAC, HVA
Naser et al. (2022)	Rats	Wister	NR/130–150 g	Rotenone (1.5 mg/kg, s.c., every 48 h for 12 days)	6/6	DMSO + Sunflower oil	p.o	80	12 days	No treatment, L-DOPA 10 mg/kg/day	GSH, NO, dopamine level, IL-6
Belviranlı et al. (2025)	Rats	Wister	4 months/450–550 g	Rotenone (2 mg/kg, s.c., daily for 35 days)	7/7	Corn oil	p.o	200	35 days	Corn oil and physiological saline, L-DOPA 10 mg/kg/day	Open field test, IL-1β, TNF-α, qRT-PCR:α-synuclein, BDNF, caspase 3, NF-κB, GAPDH
Liu et al. (2023)	Rats	Wister	6 weeks/280–320 g	6-OHDA (12 μg, stereotaxic injection into MFB/SN, single dose)	6/6	0.9% Saline (purity: >98%)	p.o	40, 80, 160	14 days	0.9% saline, L-DOPA 5 mg/kg/day	Open field test, rotarod test
Khuwaja et al. (2011)	Rats	Wister	16 weeks/250–270 g	6-OHDA (10 μg, stereotaxic injection into striatum, single dose)	8/8	1% CMC +1% Tween-80 in PBS	p.o	80	21 days	No treatment, No positive control	Rotarod test, beam-crossing task, GSH, CAT, SOD, MDA
Xia et al. (2016)	Mice	C57BL/6	8 weeks/23–25 g	MPTP (30 mg/kg/day, i.p., daily for 7 days)	6/6	NR	i.p	50	7 days	No treatment, No positive control	SOD, CAT, DOPAC, HVA, dopamine level, MDA
Alves et al. (2024)	Mice	Swiss albino	3 months/40–50 g	Rotenone (1 mg/kg, i.p., once daily for 23 days)	7/7	Soybean oil	p.o	50	30 days	No treatment, No positive control	Open field test, rotarod test, beam-crossing task, MDA, CAT
Bhowmick et al. (2022)	Rats	Wister	14 weeks/170 ± 10 g	6-OHDA (15 μg, stereotaxic injection into SNc, single dose)	8/8	Sterile water	p.o	100	84 days	No treatment, No positive control	Open field test, rotarod test
RAJESWARI. (2006)	Mice	Swiss albino	NR/NR	MPTP (4 × 10 mg/kg at 1-h intervals, i.p., total 40 mg/kg)	8/8	DMSO	p.o	80	7 days	No treatment, No positive control	GSH, SOD, CAT
Pan et al. (2007)	Mice	C57BL/6	8 weeks/20 g	MPTP (30 mg/kg, i.p., daily for 5 days)	10/10	DMSO	i.p	5, 50, 150	7 days	Physiological saline, No positive control	The cell number of TH, WB: TH, GFAP, iNOS

Abbreviations: SOD, superoxide Dismutase; GSH, glutathione; TNF-α, tumor Necrosis Factor-alpha; IL-1β, Interleukin-1, beta; NO, nitric oxide; MDA, malondialdehyde; CAT, catalase; DOPAC, 3,4-Dihydroxyphenylacetic Acid; HVA, homovanillic acid; IL-6, Interleukin-6; NA: not available; Purity information was extracted where available. “NR” indicates that the original study did not specify the purity or source of the compound; n (C/E): number of animals in control and experimental groups.

### 3.3 Quality assessment and risk of bias

**FIGURE 2:**
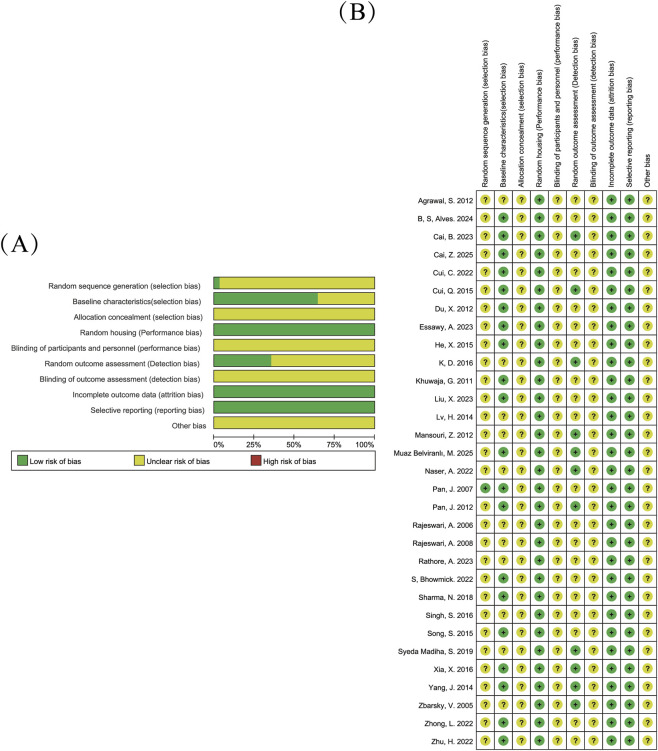
Bias risk assessment. Note: Risk of bias graph **(A)** Risk of bias summary **(B)**. Yellow indicates unclear risk of bias, and red indicates high risk of bias.

The results of the quality assessment are shown in (Figure 2). Among the 31 studies, only one study reported using a “random number table” for group allocation, while the remaining studies only mentioned random allocation without specifying the method, leading to an unclear risk rating. Eleven studies did not report the weight or age of the animals, resulting in an unclear risk rating. Regarding allocation concealment, none of the studies specified whether blinding was employed, leading to an unclear risk rating. All studies employed random allocation. None of the studies specified whether the personnel were blinded to the animal groupings, leading to an unclear risk rating. In terms of random outcome assessment, 11 studies were classified as low risk, while the rest had unclear risk ratings. For detection bias, all studies were considered to have unclear risk ratings. All studies reported complete data on the animals, and all studies reported the expected outcomes. In the “other bias” category, all studies were rated as having an unclear risk.

### 3.4 Meta-analysis

#### 3.4.1 Effects on motor function

##### 3.4.1.1 Open field test

**FIGURE 3:**
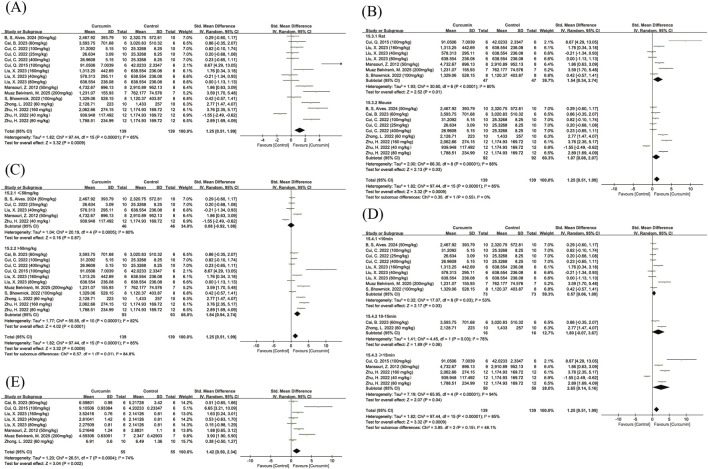
Forest plot showing the effect of curcumin on locomotor activity in the open field test. Note: Forest plots show standardized mean differences (SMDs) with 95% confidence intervals (CIs) comparing curcumin-treated groups with control groups. A random-effects model was applied when substantial heterogeneity was present. Forest plot of total movement distance in the open field test **(A)** Subgroup analysis of animal species in the open field test for total movement distance **(B)** Dose subgroup analysis in the open field test for total movement distance **(C)** Subgroup analysis based on measurement time in the open field test for total movement distance **(D)** Forest plot of average velocity in the open field test **(E)**. Negative SMD values indicate a reduction in outcome measures, whereas positive SMD values indicate an increase relative to controls. The same statistical approach was applied to all subsequent forest plots unless otherwise specified.

A total of 10 studies reporting total movement distance in the open field test were included, three of which applied different curcumin dose groups. The pooled analysis showed that, compared with the control group, curcumin treatment significantly improved locomotor activity (SMD = 1.25, 95% CI: 0.51, 1.99, Z = 3.32, P = 0.0009). However, there was high heterogeneity among the included studies (I^2^ = 85%) (Figure 3), and therefore a random-effects model was adopted.

Subgroup analysis based on animal species indicated that the effect size was slightly larger in rats than in mice, although the between-subgroup difference was not statistically significant (P = 0.55) (Figure 3B), and high heterogeneity persisted in both subgroups. When 50 mg/kg was used as the cutoff dose for subgrouping, both low- and high-dose subgroups showed high heterogeneity; nevertheless, the effect of curcumin on locomotor activity differed significantly between dose subgroups (Chi^2^ = 6.57, df = 1, P = 0.01) (Figure 3C), with a larger effect size observed in the higher-dose subgroup.

Because the included studies used different recording durations in the open field test, we further performed subgroup analyses according to testing time, categorizing studies into <10 min, 10–15 min, and >15 min groups. In the <10 min subgroup, curcumin exerted a significant pro-locomotor effect, and heterogeneity was relatively low (I^2^ = 53%) (Figure 3D). The >15 min subgroup also showed a significant promotive effect on locomotor activity, but with high between-study heterogeneity. The 10–15 min subgroup included only two studies, which suggested a relatively large effect size that did not reach statistical significance and was accompanied by high heterogeneity. No statistically significant differences were detected between these time-based subgroups.

To further assess the impact of curcumin on locomotor activity, we conducted a meta-analysis of the average movement speed in the open field test. A total of six studies were included, with one study applying different dose groups. The results showed that the curcumin treatment group exhibited a significant improvement in average velocity (SMD = 1.42, 95% CI: 0.50, 2.34, Z = 3.04, P = 0.002), although there was moderate heterogeneity (I^2^ = 74%) (Figure 3E). Given the small number of included studies, and to avoid reducing statistical power through excessive subgrouping, no subgroup analyses were performed for dose, species, or testing time.

##### 3.4.1.2 Rotarod test

**FIGURE 4:**
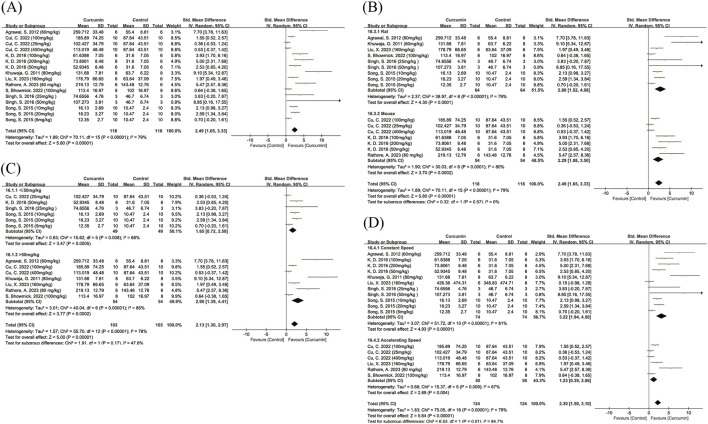
Forest plot of latency to fall in the rotarod test. Note: Forest plot of latency to fall in the rotarod test **(A)** Subgroup analysis of animal species in the rotarod test for latency to fall **(B)** Dose subgroup analysis in the rotarod test for latency to fall **(C)** Subgroup analysis based on accelerating mode in the rotarod test for latency to fall **(D)**. Positive SMD values indicate prolonged latency to fall, reflecting improved motor coordination.

A total of nine studies reporting latency to fall in the rotarod test were included, with four studies applying different dose groups. The results showed that, compared with the control group, the curcumin treatment group exhibited a significant increase in the time spent on the rotarod (SMD = 2.49, 95% CI: 1.65, 3.33, Z = 5.80, P < 0.00001), although there was high heterogeneity among the studies (I^2^ = 79%) (Figure 4A), so a random-effects model was used for analysis.

Subgroup analyses based on animal species, dose, and rotarod movement patterns showed that curcumin significantly improved motor coordination in both rats and mice. The effect size in rats was slightly higher, but the difference was not statistically significant, and high heterogeneity was present in both subgroups (Figure 4B). Dose subgroup analysis showed that both high and low doses of curcumin significantly improved motor coordination, although the high-dose group showed a larger effect size, the differences between the subgroups were not statistically significant, and high heterogeneity was observed (Figure 4C).

Based on the movement mode of the rotarod, studies were divided into constant-speed and accelerating modes for subgroup analysis. The results indicated that the curcumin treatment group significantly extended the time spent on the rotarod in both movement modes. In particular, curcumin exhibited a more significant improvement in the constant-speed mode, with the difference between the two subgroups being statistically significant (Chi^2^ = 6.53, df = 1, P = 0.01) (Figure 4D). Notably, the accelerating mode subgroup showed lower heterogeneity (I^2^ = 67%) compared with the constant-speed mode.

##### 3.4.1.3 Pole test

**FIGURE 5:**
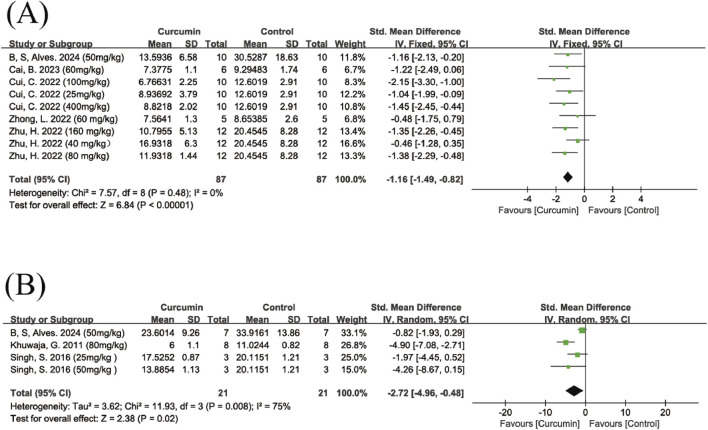
Forest plot of the pole test and balance beam test. Note: Forest plot of the pole test **(A)** Forest plot of the balance beam test **(B)**. Forest plot of the balance beam test, in which negative SMD values indicate reduced traversal time, reflecting improved balance and motor performance.

A total of five studies assessing the effect of curcumin on motor coordination in animals using the pole test were included, with two studies applying different dose groups. The results showed that the curcumin treatment group exhibited a significant improvement compared to the control group (SMD = −1.16, 95% CI: −1.49, −0.82, Z = 5.80, P < 0.00001), with low heterogeneity among the studies (I^2^ = 0%) (Figure 5A), so a fixed-effects model was used for analysis. Due to the limited number of studies included, no further subgroup analyses were performed.

##### 3.4.1.4 Balance beam test

A total of three studies reporting balance beam traversal time were included, with one study applying different dose groups. The pooled analysis showed that, compared with the control group, curcumin treatment significantly reduced the time required for animals to traverse the balance beam (SMD = −2.27, 95% CI: −4.96, −0.48, Z = 2.38, P = 0.02), although heterogeneity was high (I^2^ = 75%) (Figure 5B). After excluding one study (Khuwaja et al., 2011), heterogeneity decreased substantially (I^2^ = 25%) (Supplementary Figure S1), and the curcumin dose used in that study was markedly higher than in the others. Due to the limited number of studies, no further subgroup analyses were performed.

#### 3.4.2 Dopaminergic system and neurotransmitter biomarkers

##### 3.4.2.1 TH^+^ cell count

**FIGURE 6:**
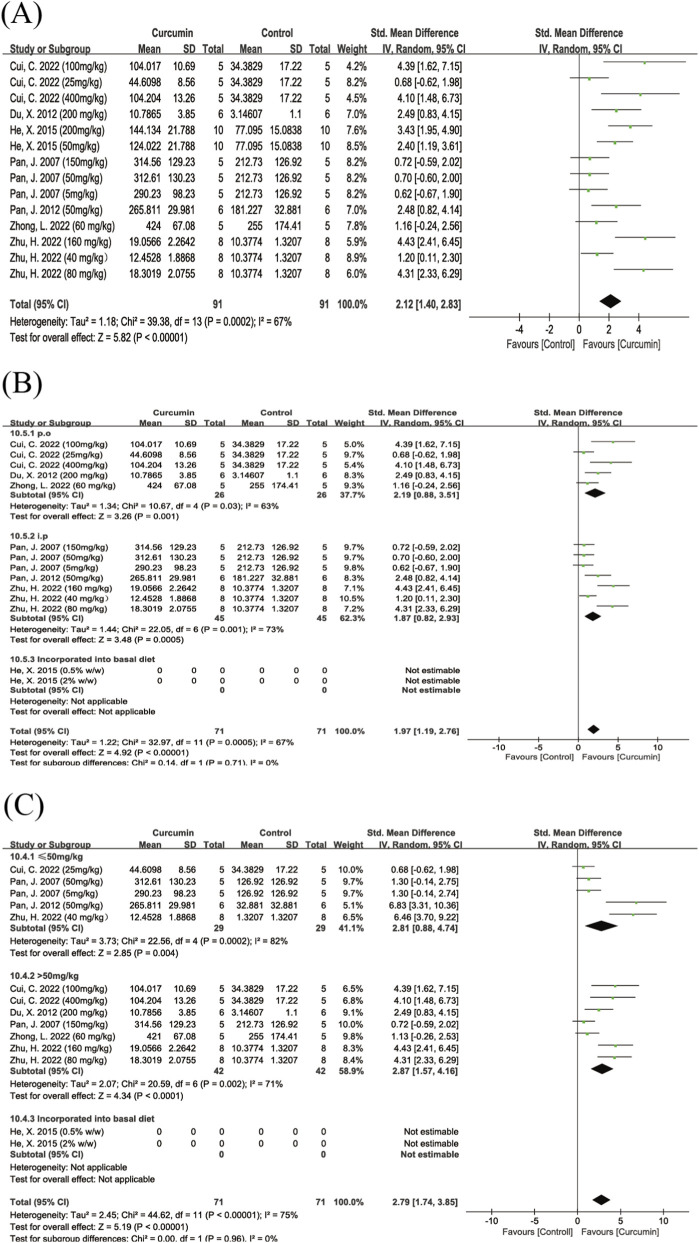
Forest plot showing the effect of curcumin on tyrosine hydroxylase-positive (TH^+^) cell counts. Note: Forest plot of tyrosine hydroxylase-positive (TH^+^) cell counts **(A)** Subgroup analysis of TH^+^ cell counts by route of administration **(B)** Dose subgroup analysis of TH^+^ cell counts **(C)** Positive SMD values indicate increased numbers of TH^+^ dopaminergic neurons relative to controls.

A total of seven studies evaluated the effect of curcumin on the number of tyrosine hydroxylase-positive (TH^+^) cells. The pooled results showed that the number of TH^+^ cells in the curcumin-treated groups was significantly higher than that in the control groups (SMD = 2.12, 95% CI: 1.40, 2.83, Z = 5.82, P < 0.00001). There was moderate heterogeneity among the included studies (I^2^ = 67%) (Figure 6A); therefore, a random-effects model was applied.

Further subgroup analyses were conducted according to the route of administration and dosage. Subgroup analysis by administration route showed that, compared with the control group, curcumin significantly increased the number of TH^+^ dopaminergic neurons regardless of the delivery method. Both intragastric and intraperitoneal administration were associated with significant neuroprotective effects, with moderate heterogeneity observed in the gavage subgroup (I^2^ = 63%) and the intraperitoneal subgroup (I^2^ = 73%). No statistically significant difference was detected between the two administration routes (Figure 6B). Subgroup analysis based on dosage indicated that both low- and high-dose curcumin treatments significantly increased the number of TH^+^ dopaminergic neurons. High heterogeneity was observed in the low-dose subgroup (I^2^ = 82%), whereas the high-dose subgroup showed moderate heterogeneity (I^2^ = 71%). No statistically significant differences were identified between the dosage subgroups. Owing to insufficient data, studies using dietary curcumin supplementation could not be included in the subgroup analyses of dosage or administration route (Figure 6C).

In addition, two other studies (Zbarsky et al., 2005; Essawy et al., 2023) also reported outcomes related to TH-positive cells; however, their results were expressed as percentages relative to a blank control group, which could not be quantitatively pooled with the other studies and were therefore not included in the meta-analysis. Nonetheless, both studies demonstrated that curcumin markedly ameliorated the loss of TH-positive neurons, which is consistent with the overall trend of our findings.

##### 3.4.2.2 Dopamine level

**FIGURE 7:**
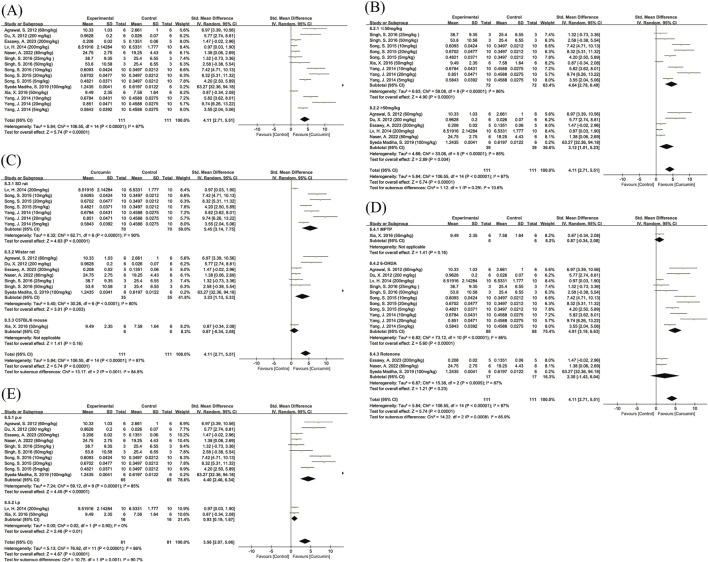
Forest plot showing the effect of curcumin on dopamine levels. Note: Forest plot of dopamine levels **(A)** Dose subgroup analysis of dopamine levels **(B)** Animal species subgroup analysis of dopamine levels **(C)** Subgroup analysis by modeling method for dopamine levels **(D)** Subgroup analysis by route of administration for dopamine levels **(E)**. Positive SMD values indicate increased dopamine levels relative to controls.

A total of 10 studies reported dopamine levels, including three studies with multiple dose groups. Pooled analysis showed that curcumin treatment significantly increased dopamine levels compared with the control group (SMD = 4.11, 95% CI: 2.71, 5.51, Z = 5.74, P < 0.00001), with substantial heterogeneity among the included studies (I^2^ = 87%) (Figure 7A). Therefore, a random-effects model was used for meta-analysis. Subgroup analyses were further performed according to dose, route of administration, animal species, and modeling method. Dose-based subgroup analysis indicated that both low-dose and high-dose curcumin groups exhibited high heterogeneity (I^2^ = 86% and 85%, respectively) (Figure 7B). In the animal species subgroup analysis, both SD rats and Wistar rats showed high heterogeneity (Figure 7C), while C57BL/6 mice were represented by only one study and thus were not suitable for subgroup comparison. Subgroup analysis by modeling method revealed high heterogeneity in both the 6-OHDA and rotenone models (Figure 7D), whereas the MPTP model was assessed in only one study and was not included in subgroup analysis.

In the route-of-administration subgroup analysis, heterogeneity was markedly reduced in the intraperitoneal injection subgroup (I^2^ = 0%) (Figure 7E); however, the pooled effect estimate did not reach statistical significance, and the number of studies was very limited.

##### 3.4.2.3 HVA

**FIGURE 8:**
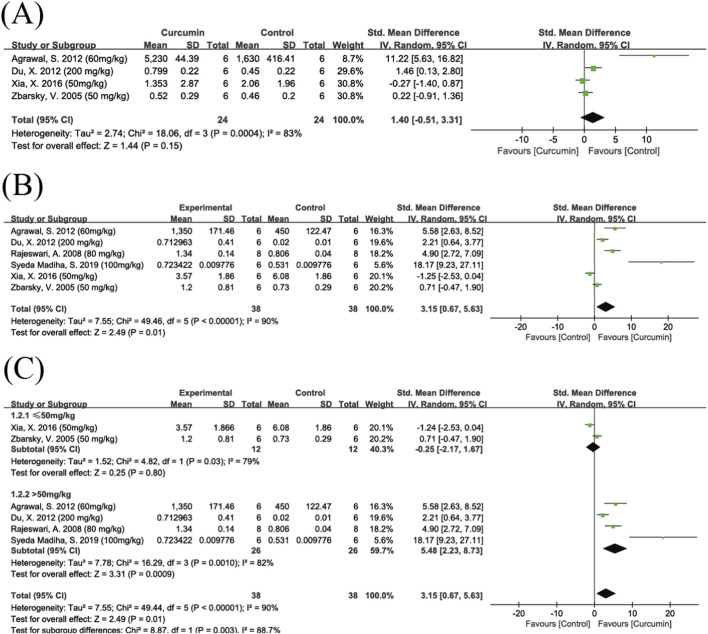
Forest plot of curcumin’s effect on HVA and DOPAC levels. Note: Forest plot of HVA levels **(A)** Forest plot of DOPAC levels **(B)** Dose subgroup analysis of DOPAC levels **(C)**. Positive SMD values indicate increased metabolite levels relative to controls.

A total of four studies evaluated the effect of curcumin on HVA levels in the brain. There was high heterogeneity among the studies (I^2^ = 83%) (Figure 8A). The results showed that the overall effect of curcumin on increasing HVA levels did not reach statistical significance (SMD = 1.40, 95% CI: −0.51, 3.31, Z = 1.44, P = 0.15). Some studies showed a significant increasing trend, while others (Xia et al., 2016) did not observe any notable changes.

##### 3.4.2.4 DOPAC

A total of six studies investigated the effect of curcumin on DOPAC levels. The pooled results showed that curcumin treatment significantly increased DOPAC levels compared to the control group (SMD = 3.15, 95% CI: 0.67, 5.63, Z = 2.49, P = 0.01) (Figure 8B). Heterogeneity analysis revealed high heterogeneity among the studies (I^2^ = 90%). Due to the small number of studies, only a dose subgroup analysis was performed.

The results indicated that low-dose curcumin treatment (≤50 mg/kg) did not significantly increase DOPAC levels, while high-dose curcumin treatment (>50 mg/kg) significantly elevated DOPAC levels (Figure 8C). The difference between the two subgroups was statistically significant (Chi^2^ = 8.87, df = 1, P = 0.003).

#### 3.4.3 Inflammatory markers

##### 3.4.3.1 IL-6

**FIGURE 9:**
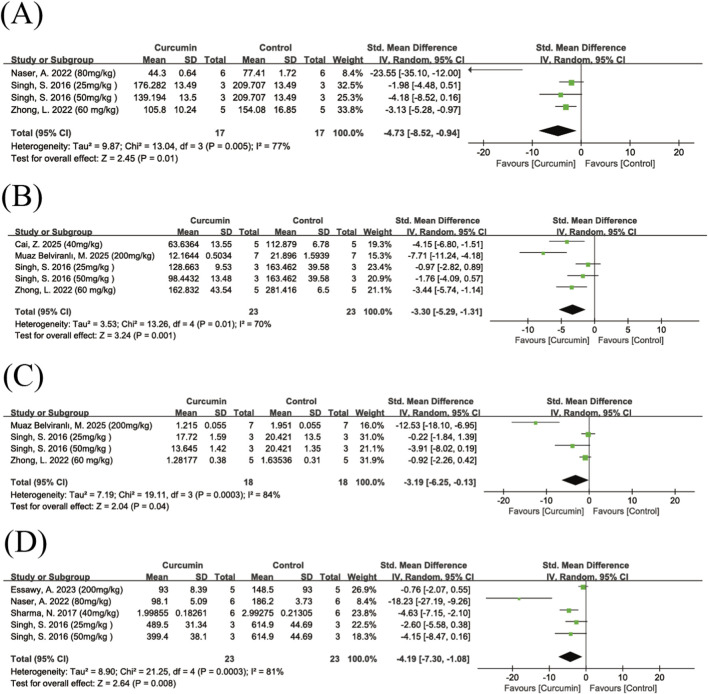
Forest plot of curcumin’s effect on inflammatory markers. Note: Forest plot of IL-6 levels **(A)** Forest plot of IL-1β levels **(B)** Forest plot of TNF-α levels **(C)** Forest plot of NO levels **(D)**. Negative SMD values indicate reduced inflammatory marker levels relative to controls.

A total of four studies investigated the effect of curcumin on IL-6 levels. The pooled results showed that curcumin significantly reduced IL-6 levels (SMD = −4.73, 95% CI: −8.52, −0.94, Z = 2.45, P = 0.01), with high heterogeneity among the studies (I^2^ = 77%) (Figure 9A). After excluding one study (Naser et al., 2022), the remaining three studies showed homogeneity (I^2^ = 0%) (Supplementary Figure S2A) and the pooled effect estimate remained statistically significant. Compared with the other included studies, this study had different dose, modeling method, and route of administration compared to the others. Due to the small number of studies, no subgroup analysis was conducted.

##### 3.4.3.2 IL-β

A total of five studies investigated the effect of curcumin on IL-1β levels. The pooled results showed that curcumin significantly reduced IL-1β levels (SMD = −3.30, 95% CI: −5.29, −1.31, Z = 3.24, P = 0.001) (Figure 9B), with moderate heterogeneity (I^2^ = 70%). After excluding one study (Belviranlı et al., 2025), heterogeneity decreased substantially (I^2^ = 40%) (Supplementary Figure S2B), and the overall effect remained statistically significant. Compared with the other included studies, this was the only study that used a high dose of curcumin (200 mg/kg).

##### 3.4.3.3 TNF-α

A total of three studies investigated the effect of curcumin on TNF-α levels. The pooled results showed that curcumin significantly reduced TNF-α levels (SMD = −3.19, 95% CI: −6.25, −0.13, Z = 2.04, P = 0.04), although there was high heterogeneity (I^2^ = 84%) (Figure 9C). After excluding one study (Belviranlı et al., 2025), heterogeneity decreased markedly (I^2^ = 27%), but the overall effect was no longer statistically significant (P = 0.16) (Supplementary Figure S2C). Compared with the other included studies, this study was the only one that used a high dose of curcumin (200 mg/kg).

##### 3.4.3.4 NO

A total of four studies investigated the effect of curcumin on NO levels. The pooled results showed that curcumin significantly reduced NO levels (SMD = −4.91, 95% CI: −7.30, −1.08, Z = 2.64, P = 0.008), with high heterogeneity (I^2^ = 81%) (Figure 9D). Due to the limited number of studies, no subgroup analysis was performed.

#### 3.4.4 Oxidative stress markers

##### 3.4.4.1 SOD

**FIGURE 10:**
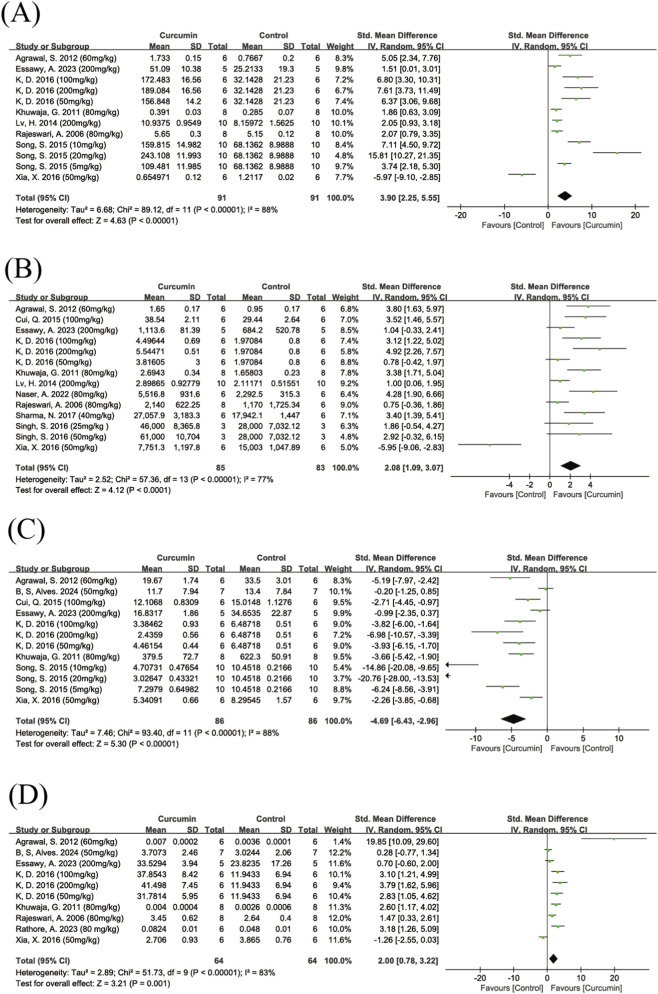
Forest plot of curcumin’s effect on oxidative stress markers. Note: Forest plot of SOD levels **(A)** Forest plot of GSH levels **(B)** Forest plot of MDA levels **(C)** Forest plot of CAT levels **(D)**. Positive SMD values indicate increased antioxidant marker levels (SOD, GSH, CAT), whereas negative SMD values indicate reduced MDA levels relative to controls.

A total of eight studies reported the effect of curcumin on SOD levels. The pooled results showed that curcumin significantly increased SOD levels (SMD = 3.90, 95% CI: 2.25, 5.55, Z = 4.63, P < 0.00001), although there was high heterogeneity among the studies (I^2^ = 88%) (Figure 10A). Subgroup analysis by route of administration revealed high heterogeneity in both the intragastric and intraperitoneal injection subgroups. Intragastric administration significantly elevated SOD levels, whereas intraperitoneal injection did not show a significant effect. The difference between the two administration routes was statistically significant (Chi^2^ = 8.85, df = 1, P = 0.003). Subgroup analyses by dose and animal species also showed high heterogeneity, with no significant between-subgroup differences (Supplementary Figures S3A,B).

Furthermore, one study (Xia et al., 2016) showed results in the opposite direction compared to the other studies. The subgroups to which this study belonged (intraperitoneal injection, low dose, rats) did not demonstrate a significant effect. A review of the original article revealed no obvious differences in administration route, dose, animal species, modeling method, or experimental procedures compared to the other studies. After excluding this study with opposite results, overall heterogeneity decreased slightly but remained at a high level, indicating that this study may have contributed partially to the heterogeneity (Supplementary Figure S3C).

##### 3.4.4.2 GSH

A total of 11 studies reported the effect of curcumin on GSH levels. The pooled results showed that curcumin significantly increased GSH levels (SMD = 2.08, 95% CI: 1.09, 3.07, Z = 4.12, P < 0.0001), although there was high heterogeneity among the studies (I^2^ = 77%) (Figure 10B). After excluding one study (Xia et al., 2016) that showed results in the opposite direction compared to the other studies, overall heterogeneity decreased slightly (I^2^ = 64%) (Supplementary Figure S4A). Subgroup analyses by dose, route of administration, and animal species were performed, and the results showed that all subgroups exhibited a certain degree of heterogeneity (Supplementary Figures S3A,B).

##### 3.4.4.3 MDA

A total of eight studies reported the effect of curcumin on MDA levels. The pooled results showed that curcumin significantly reduced MDA levels (SMD = −4.69, 95% CI: −6.43, −2.96, Z = 5.30, P < 0.0001) (Figure 10C), although there was high heterogeneity among the studies (I^2^ = 88%). Subgroup analyses showed no statistically significant differences between subgroups based on dose or animal species (Supplementary Figure S5A,B). As only one study used intraperitoneal injection, no subgroup analysis by route of administration was performed.

##### 3.4.4.4 CAT

A total of eight studies reported the effect of curcumin on CAT levels. The pooled results showed that curcumin significantly increased CAT levels (SMD = 2.00, 95% CI: 0.78, 3.22, Z = 3.21, P = 0.001) (Figure 10D), although there was high heterogeneity among the studies (I^2^ = 83%). Dose-based subgroup analysis showed that heterogeneity remained high in both the low-dose and high-dose subgroups; the low-dose subgroup did not show a statistically significant effect, whereas the high-dose subgroup showed a significant increase in CAT levels. Due to the limited number of studies, subgroup analyses based on route of administration and animal species were not performed.

### 3.5 Publication bias

**FIGURE 11:**
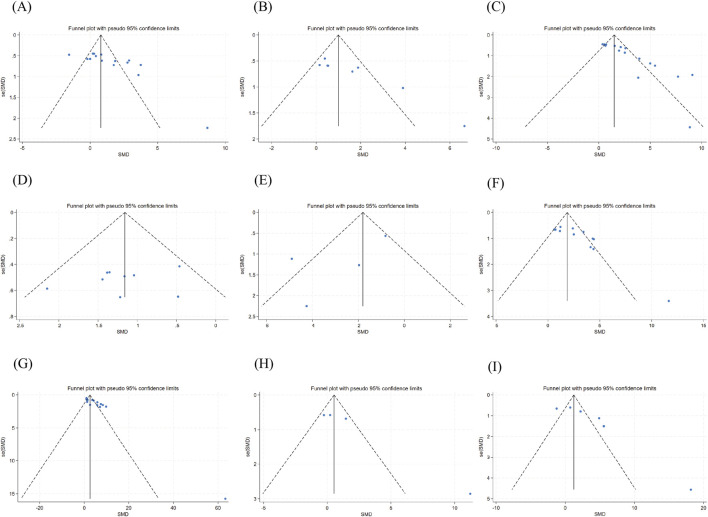
Funnel plots for publication bias assessment. Note: Funnel plots display standardized mean differences (SMDs) plotted against their standard errors for visual assessment of potential publication bias. Symmetry of the funnel plots suggests a low risk of publication bias, whereas asymmetry may indicate potential small-study effects. Funnel plot of total movement distance in the open field test **(A)** Funnel plot of average movement speed in the open field test **(B)** Funnel plot of Rotarod test **(C)** Funnel plot of Pole descent latency **(D)** Balance Beam Test **(E)** TH^+^ cell counts **(F)** DA **(G)** HVA **(H)** DOPAC **(I)**.

Funnel plots and Egger’s tests for motor function and dopaminergic markers are presented in (Figure 11) and (Table 2), respectively. Additional results are available in Supplementary Figure S6 and Supplementary Table S1 Except for CAT, dopamine, TH^+^ cell count, and the pole test, visual inspection of the funnel plots for the remaining outcome measures showed obvious asymmetry in data distribution. Egger’s test further confirmed the presence of statistical evidence for publication bias in all outcome measures except HVA, IL-6, TNF-α, SOD, GSH, pole test, and balance beam traversal time (P < 0.05). However, for some outcome measures, the number of included studies was relatively small (<10), which may have compromised the robustness of the publication bias assessment. Therefore, the relevant conclusions should be interpreted with caution.

**TABLE 2:** Egger’s regression test for assessment of publication bias.

Outcome	t	p	95% conf. Interval		No. of studies (containing different doses)
Total movement distance in the open field test	4.05	0.001	3.182998	10.35191	16
Average movement speed in the open field test	5.39	0.002	2.992676	7.962022	8
Rotarod test	7.06	0.000	2.726074	5.102994	16
Pole descent latency	−0.75	0.475	−7.236673	3.736939	9
Balance beam test	−1.51	0.270	−10.82985	5.199256	4
TH^+^ cell counts	4.64	0.000	2.437204	6.690423	15
DA	6.07	0.000	3.245724	6.832594	15
HVA	4.16	0.053	−0.169866	10.3363	4
DOPAC	3.48	0.025	1.273341	11.39212	6

Egger’s regression test was used to statistically assess potential publication bias for each outcome. P value <0.05 was considered indicative of significant funnel plot asymmetry. The 95% confidence interval refers to the intercept estimate from the regression analysis. No. of studies indicates the number of independent comparisons included, accounting for studies with multiple dose groups. Abbreviations: DA: dopamine level; HVA: homovanillic acid; DOPAC, 3,4-Dihydroxyphenylacetic Acid.

### 3.6 Sensitivity analysis

**FIGURE 12:**
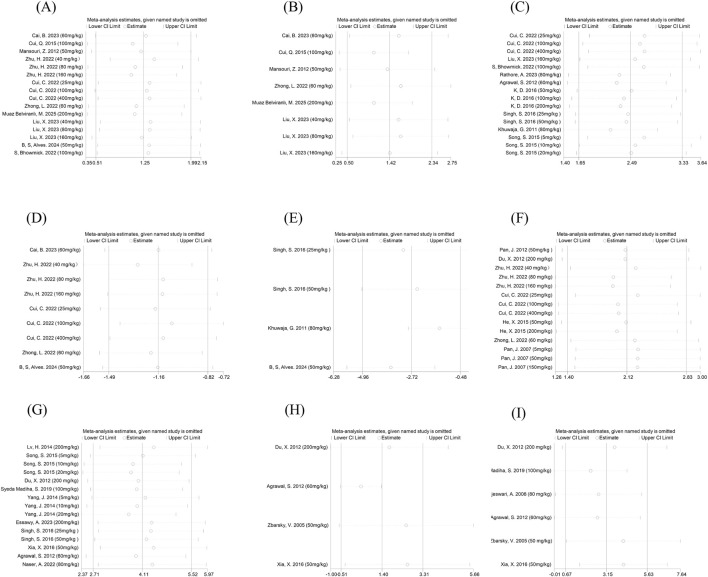
Outcome indicators sensitivity analysis. Note: Sensitivity analyses were conducted using a leave-one-out approach, in which individual studies were sequentially removed to assess the robustness of pooled effect estimates. Forest plots display standardized mean differences (SMDs) with 95% confidence intervals (CIs) recalculated after omission of each study. Consistency of effect estimates across iterations suggests the stability of the pooled results; total movement distance in the open field test **(A)** average movement speed in the open field test **(B)** latency to fall in the rotarod test **(C)** Pole descent latency **(D)** balance beam traversal time **(E)** TH^+^ cell counts **(F)** DA **(G)** HVA **(H)** DOPAC **(I)**.

To assess the robustness of our findings, we conducted sensitivity analyses across all outcome measures by systematically excluding individual studies. The analysis revealed remarkable consistency: removing any single study produced negligible alterations in both the pooled effect estimates and their corresponding confidence intervals, thereby confirming the stability and reliability of our meta-analytic results. Sensitivity analyses for motor function outcomes and dopaminergic markers are illustrated in (Figure 12), whereas other outcomes are detailed in (Supplementary Figure S7).

## 4 Discussion

### 4.1 Summary of evidence

**FIGURE 13:**
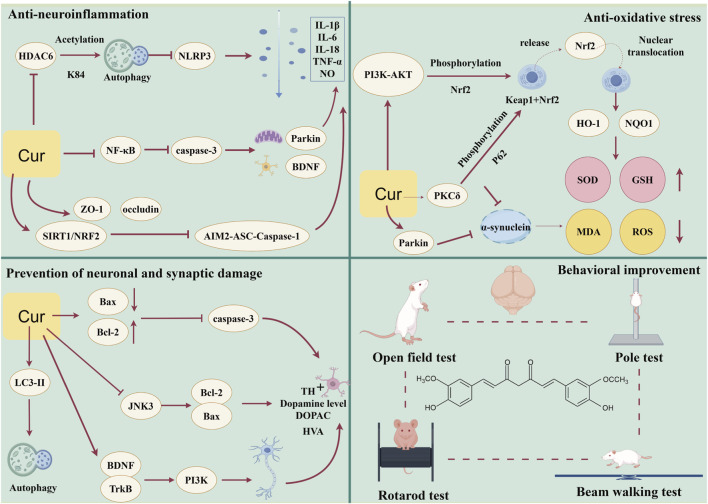
Schematic illustration of the potential mechanisms by which curcumin may ameliorate Parkinson’s disease. Based on preclinical evidence, Curcumin is proposed to exert neuroprotective effects through pleiotropic mechanisms, including suppression of neuroinflammation, attenuation of oxidative stress, facilitation of α-synuclein clearance, and maintenance of neuronal integrity.

This meta-analysis included 31 studies involving 552 PD animal models, all published in English. Overall, the evidence suggests that curcumin exerts a broad neuroprotective profile, encompassing improvements in motor performance, preservation of dopaminergic neuronal integrity, and modulation of neuroinflammatory and oxidative stress–related processes. Rather than acting through a single pathway, curcumin appears to confer neuroprotection via multi-target mechanisms that collectively support dopaminergic function and neural homeostasis. However, these findings should be interpreted with caution. Substantial heterogeneity was observed across several outcome measures, likely reflecting variability in animal species, disease models, dosing regimens, routes of administration, and treatment duration. Additionally, heterogeneity may also stem from limited characterization of the administered curcumin. Only a small proportion of the included studies explicitly reported compound purity, while most did not provide detailed information regarding material characterization, which may influence pharmacokinetic properties and biological efficacy. Moreover, the included studies investigated both purified curcumin and curcumin-rich extracts, which are not strictly pharmacologically equivalent. Although these interventions share curcuminoid-related mechanistic rationales, their compositional differences may further contribute to inter-study heterogeneity and warrant cautious interpretation of the pooled results. In addition, methodological limitations were common among the included studies, particularly insufficient reporting of randomization, allocation concealment, and blinding, which may have introduced bias and contributed to effect size inflation. These factors limit the certainty of the pooled estimates and underscore the need for more standardized and rigorously designed animal studies. Importantly, all mechanistic insights discussed in this review are derived exclusively from preclinical evidence, and their translational relevance to clinical Parkinson’s disease remains to be established (Figure 13).

Interestingly, while curcumin consistently preserved TH^+^ dopaminergic neurons and increased striatal dopamine levels across studies, its effects on downstream dopamine metabolites such as DOPAC and HVA were less consistent. This discrepancy may reflect differential sensitivity of neuronal integrity versus dopamine metabolism to curcumin intervention, as well as greater methodological variability in metabolite quantification. These findings suggest that curcumin’s neuroprotective effects may be more robust at the level of dopaminergic neuron preservation than at the level of dopamine turnover. Notably, subgroup analysis suggested that intragastric administration of curcumin was associated with a larger effect size on dopamine levels than intraperitoneal administration. However, given the limited number of studies included in this subgroup, this finding should be interpreted with caution. Importantly, this pattern does not necessarily indicate superior systemic bioavailability of orally administered curcumin, which is known to exhibit poor absorption and extensive first-pass metabolism (Anand et al., 2007). Rather, first-pass metabolism may generate bioactive curcumin metabolites that contribute to neuroprotection (Nelson et al., 2017). In addition, intragastric administration may result in sustained, low-level systemic exposure rather than transient peak plasma concentrations. Given that orally administered curcumin demonstrates biological activity despite low systemic bioavailability, it has been suggested that its therapeutic effects may not be strictly dependent on high peak concentrations, but may instead relate to prolonged exposure and gastrointestinal-related mechanisms (Lopresti, 2018). Furthermore, oral delivery directly engages the gastrointestinal tract and may modulate peripheral inflammation and gut–brain axis signaling, which have been increasingly implicated in dopaminergic neuron survival and Parkinson’s disease pathogenesis (Houser and Tansey, 2017). Collectively, differences in pharmacokinetic profiles and biological pathways activated by distinct administration routes may partially explain the observed heterogeneity in neuroprotective outcomes. However, direct pharmacokinetic–pharmacodynamic comparisons between administration routes in Parkinson’s disease models remain limited, and further well-designed experimental studies are warranted to clarify these mechanisms.

In addition, although curcumin consistently modulated inflammatory cytokines and oxidative stress markers across studies, substantial heterogeneity was observed for several biochemical outcomes. This variability likely reflects differences in experimental conditions, biomarker selection, and timing of outcome assessment, rather than fundamental inconsistencies in the direction of curcumin’s anti-inflammatory and antioxidant effects. Several subgroup analyses indicated differential effects between low- and high-dose curcumin interventions for selected outcomes; however, these findings should be interpreted cautiously due to limited study numbers and persistent heterogeneity, and do not allow definitive conclusions regarding dose–response relationships.

### 4.2 Inflammatory mechanisms

Inflammation plays a critical role in the pathological progression of neurodegenerative diseases. Previous studies have detected significantly elevated levels of inflammatory factors in both brain tissue and peripheral blood of patients with PD. In the pathogenesis of PD, excessive uptake of abnormally aggregated α-synuclein (α-syn) by dopaminergic neurons activates microglia and induces the release of pro-inflammatory cytokines, triggering a sustained inflammatory stress response that ultimately leads to neuronal apoptosis (Alvarez-Erviti et al., 2011). A natural product with well-characterized anti-inflammatory and multi-target properties, reinforcing the network pharmacology approach. This supports the concept that multi-target natural products like curcumin hold promise for modulating complex neuroinflammatory cascades in neurodegenerative diseases (Wang et al., 2024).

The anti-inflammatory mechanisms of curcumin involve the modulation of multiple signaling pathways. Studies have shown (Belviranlı et al., 2025) that curcumin can inhibit the expression of hippocampal nuclear factor kappa B (NF-κB), reduce serum levels of pro-inflammatory factors such as TNF-α and IL-1β, downregulate the expression of the pro-apoptotic factor caspase-3, upregulate the mitochondrial autophagy marker parkin, increase brain-derived neurotrophic factor (BDNF) levels, and reduce pathological aggregation of α-synuclein, thereby ameliorating PD-related motor dysfunction, depressive-like behavior, and cognitive memory impairment. Furthermore, Cai et al. (Cai et al., 2025)revealed that curcumin exerts neuroprotective effects by inhibiting the HDAC6-NLRP3 signaling pathway. Specifically, curcumin inhibits HDAC6, maintaining high acetylation of NLRP3 at the K84 site. This acetylation modification serves as a “degradation signal” that promotes NLRP3 clearance through the autophagy pathway, thereby reducing the overall level of the NLRP3 inflammasome and decreasing the release of downstream pro-inflammatory factors IL-1β, Interleukin-18 (IL-18), and cleaved caspase-1, ultimately attenuating inflammatory damage to substantia nigra dopaminergic neurons and improving α-synuclein aggregation and dopaminergic neuronal loss.

Notably, curcumin can also exert neuroprotective effects through the gut-brain axis. Zhong et al. (Zhong et al., 2022) demonstrated that curcumin activates the SIRT1/NRF2 signaling pathway in intestinal tissue, inhibiting AIM2 inflammasome-mediated pyroptosis cascade (AIM2-ASC-Caspase-1), thereby reducing the release of pro-inflammatory cytokines IL-1β, IL-6, IL-18, and TNF-α. Concurrently, curcumin upregulates the expression of intestinal tight junction proteins ZO-1 and occludin, restores intestinal barrier function, blocks the transmission of peripheral inflammatory signals to the central nervous system, and thereby attenuates neuroinflammation in the nigrostriatal system and protects dopaminergic neurons. This finding provides novel theoretical support for curcumin’s therapeutic potential in PD through modulation of peripheral immune-central nervous system interactions.

### 4.3 Oxidative stress mechanisms

Oxidative stress is one of the central pathological mechanisms in the pathogenesis of PD. Dopaminergic neurons in the substantia nigra pars compacta are particularly vulnerable to oxidative damage due to their high metabolic activity, abundant mitochondrial content, and the generation of reactive oxygen species during dopamine oxidative metabolism. In the pathological progression of PD, factors such as mitochondrial dysfunction, abnormal aggregation of α-syn, and iron metabolism dysregulation collectively contribute to excessive ROS production. When ROS generation exceeds the clearance capacity of the endogenous antioxidant defense system, oxidative stress ensues, leading to lipid peroxidation, protein oxidative damage, DNA damage, and further deterioration of mitochondrial function, ultimately resulting in selective apoptosis of dopaminergic neurons (Dong-Chen et al., 2023). CAT, SOD, and GSH work synergistically to maintain redox homeostasis, while MDA, the end product of lipid peroxidation, reflects the extent of oxidative damage.

Studies have shown (Cui et al., 2016; Song et al., 2016) that curcumin activates the PI3K/Akt signaling pathway to phosphorylate nuclear factor erythroid 2-related factor 2 (Nrf2), promoting its release from the Keap1 inhibitory complex and nuclear translocation. This leads to transcriptional activation of downstream antioxidant enzymes, including heme oxygenase-1 (HO-1) and NAD(P)H: quinone oxidoreductase 1 (NQO1), thereby reducing ROS and MDA levels, blocking ROS-mediated activation of NF-κB and the NLRP3 inflammasome, decreasing pro-inflammatory cytokine release, and breaking the vicious cycle of “oxidative stress-inflammation.” Notably, curcumin also activates protein kinase C δ (PKCδ) to promote p62 phosphorylation. Phosphorylated p62 competitively binds to Keap1, further enhancing Nrf2 activation and forming a p62-Keap1-Nrf2 positive feedback loop (Rathore et al., 2023). Simultaneously, curcumin upregulates the expression of the autophagy marker microtubule-associated protein one light chain 3-II (LC3-II), enhancing autophagic clearance of pathological α-synuclein, thereby enabling the autophagy system and antioxidant stress system to synergistically exert neuroprotective effects.

### 4.4 Dopaminergic neuron protection and α-synuclein clearance

The core pathological features of Parkinson’s disease include abnormal aggregation of α-syn, progressive loss of TH^+^ neurons in the substantia nigra pars compacta, and a significant decline in striatal dopamine levels. When TH^+^ neuronal loss exceeds 50%, the characteristic motor symptoms of PD begin to manifest (Kordower et al., 2013), accompanied by corresponding reductions in the dopamine metabolites DOPAC and HVA. Curcumin promotes α-syn clearance and protects dopaminergic neurons through multi-target and multi-pathway mechanisms (Nebrisi, 2021; Cai et al., 2025), including activation of autophagy to enhance α-syn degradation, inhibition of neuroinflammatory pathways, and suppression of ferroptosis—an iron-dependent form of programmed cell death increasingly implicated in PD pathogenesis (Foroutan et al., 2024).

At the protein quality control level, studies have shown (Belviranlı et al., 2025) that curcumin upregulates the expression of the mitochondrial autophagy marker Parkin. As an E3 ubiquitin ligase, Parkin promotes ubiquitination of α-syn, enhancing its degradation via the ubiquitin-proteasome system or the autophagy-lysosome pathway. Rathore et al. (Rathore et al., 2023) further revealed that curcumin activates PKCδ to promote phosphorylation of p62 at Ser351. Phosphorylated p62 acts as an autophagy receptor that recognizes ubiquitinated misfolded α-syn and recruits it to LC3-II-decorated autophagosomes, facilitating degradation of pathological α-syn aggregates through the autophagosome-lysosome fusion pathway.

### 4.5 Neuronal protection mechanisms

In terms of neuronal protection, curcumin exerts anti-apoptotic effects through multi-level regulation of the B-cell lymphoma 2 (Bcl-2) family proteins. At the same time, curcumin upregulates the expression of the autophagy marker LC3-II, enhancing overall autophagic flux, while simultaneously downregulating the pro-apoptotic protein Bax and upregulating the anti-apoptotic protein Bcl-2, thereby blocking the caspase-3-mediated apoptotic cascade (Rathore et al., 2023). Second, Pan et al. (Pan et al., 2012) demonstrated that curcumin reduces phosphorylation of Bcl-2 at the Ser87 site by inhibiting c-Jun N-terminal kinase 3 (JNK3) activity, maintaining the functionally active form of Bcl-2, enhancing its binding affinity to Bax, preventing Bax translocation from the cytoplasm to mitochondria, and blocking the mitochondria-mediated intrinsic apoptotic pathway. This dual regulation of “expression upregulation” and “activity maintenance” synergistically enhances the anti-apoptotic effects of Bcl-2, effectively protecting dopaminergic neurons from apoptosis. Furthermore, Yang et al. (Yang et al., 2014) elucidated the mechanism by which curcumin modulates dopamine levels through activation of the hippocampal BDNF/TrkB/PI3K signaling pathway. Curcumin upregulates the expression of BDNF and its high-affinity receptor TrkB, activates the downstream PI3K pathway, promotes neuronal survival and synaptic plasticity, and significantly elevates hippocampal dopamine and norepinephrine levels, thereby ameliorating neurotransmitter dysregulation. In addition to its direct anti-apoptotic effects, emerging evidence indicates that curcumin can precondition stem cells to enhance their neuroprotective capacity by modulating microglial phenotypes and suppressing PAN optosis, a coordinated cell death pathway involving apoptosis, necroptosis, and pyroptosis (Lan et al., 2024). This highlights curcumin’s potential not only as a direct neuroprotectant but also as a modulator of the neurogenic niche and immune cell function in neurodegenerative settings. Collectively, curcumin comprehensively protects dopaminergic neuronal function through multiple mechanisms, including promotion of α-syn clearance, enhancement of anti-apoptotic defenses, and activation of neurotrophic factor signaling pathways, providing a solid molecular foundation for its therapeutic potential in Parkinson’s disease.

### 4.6 Limitations

Despite the systematic evaluation of the pharmacological effects and mechanisms of curcumin in the treatment of Parkinson’s disease, this meta-analysis has several limitations. First, the included studies lacked standardization in experimental protocols, particularly in behavioral assessment methods, scoring criteria, and observation timepoints, which may affect the reproducibility of results and comparability across studies. Moreover, substantial heterogeneity was observed among studies. Although random-effects models were employed and subgroup analyses along with sensitivity analyses were conducted to explore sources of heterogeneity, several outcome measures still exhibited high statistical heterogeneity (I^2^ > 50%). Additionally, the limited number of studies in certain subgroups restricted the statistical power of subgroup analyses, preventing a comprehensive elucidation of how different experimental conditions (such as animal model type, dosage, and treatment duration) influence the pharmacological effects of curcumin. In addition, the inclusion of both purified curcumin and curcumin-rich extracts, which differ in compositional complexity, represents an additional source of heterogeneity and limits precise pharmacological comparability across studies. Furthermore, the poor bioavailability and inconsistent pharmacokinetic profiles of curcumin due to its low solubility and rapid metabolism represent a major translational challenge. Recent advances in formulation science, such as curcumin-loaded Pickering emulsions, have shown promise in enhancing its digestive stability, absorption, and metabolic regulation (Wang et al., 2025), suggesting that delivery optimization could reduce inter-study variability and improve therapeutic reproducibility. Therefore, future research should prioritize standardization of experimental design, increase the number of high-quality studies to reduce heterogeneity, and develop advanced delivery systems to overcome curcumin’s pharmacokinetic limitations, thereby enhancing evidence reliability and translational potential. Crucially, while these results demonstrate the significant pharmacological effects of curcumin in preclinical models, these findings should not be directly equated with clinical efficacy in human patients. Therefore, further rigorous clinical validation is essential to confirm these neuroprotective effects in a clinical setting.

Second, the included studies demonstrated notable deficiencies in quality assessment and risk of bias control. None of the included studies explicitly reported specific randomization methods, nor did they clarify whether blinding was implemented during outcome assessment, potentially introducing selection bias and detection bias that could overestimate treatment effect sizes. According to the SYRCLE risk of bias assessment tool, most studies were rated as “unclear” or “high risk” in critical domains including random sequence generation, allocation concealment, and blinding implementation, thereby limiting the credibility of the evidence. Consequently, future animal studies should strictly adhere to reporting guidelines such as ARRIVE or similar standards, explicitly describe randomization methods at the experimental design stage (e.g., random number tables, computer-generated random sequences), implement allocation concealment measures, and apply blinding to investigators during data collection and outcome assessment to minimize bias and enhance the scientific rigor and reliability of experimental findings.

Lastly, abnormal aggregation of α-syn and Lewy body formation represent key pathological hallmarks of PD. Although the majority of studies included in this meta-analysis demonstrated significant protective effects of curcumin on dopaminergic neurons, only a limited number of studies investigated the specific molecular mechanisms underlying curcumin-mediated α-syn clearance. Therefore, future research should further elucidate the direct mechanisms by which curcumin modulates α-syn pathology, including its effects on α-syn oligomers and fibrillar aggregates, its inhibitory effects on Lewy body formation, and causal validation of the associated signaling pathways, in order to more comprehensively reveal the pathological basis for curcumin’s therapeutic potential in Parkinson’s disease.

### 4.7 Safety and toxicity

Curcumin generally exhibits favorable tolerability in both clinical and experimental studies; however, it may elicit certain adverse reactions in humans or animals. A single-dose escalation study (Lao et al., 2006) in 24 healthy volunteers receiving oral doses ranging from 500 to 12,000 mg showed that approximately 30% of participants (7 individuals) experienced mild adverse events, including diarrhea, headache, rash, and yellow-colored stools. All events were Grade 1, with no serious toxicity or dose-dependent effects observed. A Phase IIA clinical trial (Carroll et al., 2011) evaluating curcumin for colorectal tumor prevention found that some participants developed diarrhea, abdominal distension, and gastroesophageal reflux symptoms after curcumin administration, though symptoms were mild and did not require discontinuation. Additionally, a case report (Imam et al., 2019) described a Caucasian woman who developed jaundice and acholic stools after self-administering high-dose curcumin supplementation (500 mg/day) as a replacement for simvastatin without physician guidance. Liver function tests revealed alanine aminotransferase (ALT) and aspartate aminotransferase (AST) elevations approximately 40% above normal limits. After discontinuation of curcumin for 42 days, the jaundice completely resolved, and liver function normalized. Furthermore, curcumin powder may induce contact allergic reactions during grinding and exposure. An occupational exposure case report (Liddle et al., 2006) documented a female pharmaceutical worker who developed erythematous rashes on the eyelids, face, neck, and hands following prolonged exposure to curcumin powder, with symptom resolution after removal from the exposure source.

Among the animal studies included in this meta-analysis, none systematically assessed the safety or toxicity of curcumin. Future research should include basic safety endpoints—such as body weight monitoring, organ weight assessment, and blood biochemistry analysis—in animal studies, while systematically investigating curcumin’s safety profile under varying doses, routes of administration, and exposure durations, and elucidating potential toxicological mechanisms to better inform translational research. Moreover, optimizing curcumin formulations to improve bioavailability while strengthening mechanistic links between natural product research and clinical applications will be crucial for advancing its therapeutic potential in neurodegenerative diseases (Hui et al., 2024).

## 5 Conclusion

This study conducted a systematic and comprehensive analysis of preclinical research on curcumin in rodent models of PD. The results demonstrated that curcumin exerts significant neuroprotective effects, effectively ameliorating motor dysfunction and neuronal damage in PD animal models. These protective effects are likely achieved through synergistic multi-mechanistic pathways, including attenuation of oxidative stress, suppression of inflammatory responses, and promotion of neuronal survival. Despite these promising preclinical findings, significant heterogeneity, potential publication bias, and a lack of standardized safety assessments in animal models highlight important limitations and the need for more rigorous preclinical studies before clinical translation. Future research should further optimize experimental design and quality control in animal studies to enhance reproducibility and comparability of results. Additionally, in-depth investigation of curcumin’s capacity to degrade α-syn and its underlying molecular mechanisms is warranted to provide more robust experimental evidence for its translational application in PD prevention and treatment.

## Data Availability

The original contributions presented in the study are included in the article/Supplementary Material, further inquiries can be directed to the corresponding author.
