# Supplementary material for: Pharmacological effects and mechanisms of curcumin in animal models of Parkinson’s disease: a systematic review and meta-analysis
Source: Front Pharmacol. 2026 Mar 9;17:1779921. doi: 10.3389/fphar.2026.1779921 (PMC13006684; doi:10.3389/fphar.2026.1779921)
Supplement: Supplementary file 1 [file Supplementaryfile1.docx]

Supplementary Material

# Supplementary Data

Search strategy in 8 Databases (EMBASE, PubMed, Web of Science, and the Cochrane Library), and they are presented respectively in the form of tables.

## Search strategy in Embase

| **Steps** | **Search terms** | **Results** |
| --- | --- | --- |
| #1 | 'parkinson disease'/exp OR 'parkinson disease' | 232211 |
| #2 | 'idiopathic parkinsonism':ti,ab,kw OR 'lewy bodies of parkinson disease':ti,ab,kw OR 'lewy bodies of parkinson`s disease':ti,ab,kw OR 'lewy bodies of parkinsons disease':ti,ab,kw OR 'lewy body parkinson disease':ti,ab,kw OR 'lewy body parkinson`s disease':ti,ab,kw OR 'lewy body parkinsons disease':ti,ab,kw OR 'paralysis agitans':ti,ab,kw OR 'parkinson dementia complex':ti,ab,kw OR 'parkinson`s disease':ti,ab,kw OR 'parkinsons disease':ti,ab,kw OR 'primary parkinsonism':ti,ab,kw OR 'parkinson disease':ti,ab,kw | 203050 |
| #3 | Search #1 OR #2 | 258112 |
| #4 | 'curcumin'/exp OR 'curcumin' | 46264 |
| #5 | 'curcuma':ti,ab,kw OR 'turmeric':ti,ab,kw OR '1,6-heptadiene-3,5-dione, 1,7-bis(4-hydroxy-3-methoxyphenyl)-, (e,e)-':ti,ab,kw OR 'turmeric yellow':ti,ab,kw OR 'yellow, turmeric':ti,ab,kw OR 'curcumine':ti,ab,kw | 12412 |
| #6 | Search #4 OR #5 | 51803 |
| #7 | Search #3 AND #6 | 1334 |

## Search strategy in PubMed

| **Steps** | **Search terms** | **Results** |
| --- | --- | --- |
| #1 | "Parkinson Disease"[Mesh] | 91162 |
| #2 | **''idiopathic parkinsonism''[Title/Abstract] OR ''lewy bodies of parkinson disease''[Title/Abstract] OR ''lewy bodies of parkinson`s disease''[Title/Abstract] OR ''lewy bodies of parkinsons disease''[Title/Abstract] OR ''lewy body parkinson disease''[Title/Abstract] OR ''lewy body parkinson`s disease''[Title/Abstract] OR ''lewy body parkinsons disease''[Title/Abstract] OR ''paralysis agitans''[Title/Abstract] OR ''parkinson dementia complex''[Title/Abstract] OR ''parkinson`s disease''[Title/Abstract] OR ''parkinsons disease''[Title/Abstract] OR ''primary parkinsonism''[Title/Abstract] OR ''parkinson disease''[Title/Abstract]** | 137172 |
| #3 | Search #1 OR #2 | 150285 |
| #4 | curcumin[MeSH Terms] | 17139 |
| #5 | (((((Curcuma[Title/Abstract]) OR (Turmeric[Title/Abstract])) OR (1,6-Heptadiene-3,5-dione, 1,7-bis(4-hydroxy-3-methoxyphenyl)-, (E,E)-[Title/Abstract])) OR (Turmeric Yellow[Title/Abstract])) OR (Yellow, Turmeric[Title/Abstract])) OR (curcumine[Title/Abstract]) | 8535 |
| 6 | Search #4 OR #5 | 22146 |
| 7 | Search 3# OR #6 | 226 |

## Search strategy in Web of Science

| **Steps** | **Search terms** | **Results** |
| --- | --- | --- |
| #1 | Parkinson Disease (Topic) OR Idiopathic Parkinson Disease (Topic) OR Idiopathic Parkinson's Disease (Topic) OR idiopathic parkinsonism (Topic) OR Lewy Body Parkinson Disease (Topic) OR Lewy Body Parkinson's Disease (Topic) OR Paralysis Agitans (Topic) OR Parkinson Disease, Idiopathic (Topic) OR Parkinson's Disease (Topic) OR Parkinson's Disease, Idiopathic (Topic) OR Parkinson's Disease, Lewy Body (Topic) OR Primary Parkinsonism (Topic) OR Parkinsonism, Primary (Topic) OR Parkinson dementia complex (Topic) and Preprint Citation Index (Exclude – Database) | 231021 |
| #2 | curcumin (Topic) OR Curcuma (Topic) OR Turmeric (Topic) OR 1,6-Heptadiene-3,5-dione, 1,7-bis(4-hydroxy-3-methoxyphenyl)-, (E,E)- (Topic) OR Turmeric Yellow (Topic) OR Yellow, Turmeric (Topic) OR curcumine (Topic) and Preprint Citation Index (Exclude – Database) | 53247 |
| #3 | #1 AND #2 | 693 |

## Search strategy in Cochrane Library

| **Steps** | **Search terms** | **Results** |
| --- | --- | --- |
| #1 | MeSH descriptor: [Parkinson Disease] explode all trees | 6338 |
| #2 | disease':ti,ab,kw OR 'lewy body parkinson disease':ti,ab,kw OR 'lewy body parkinson`s disease':ti,ab,kw OR 'lewy body parkinsons disease':ti,ab,kw OR 'paralysis agitans':ti,ab,kw OR 'parkinson dementia complex':ti,ab,kw OR 'parkinson`s disease':ti,ab,kw OR 'parkinsons disease':ti,ab,kw OR 'primary parkinsonism':ti,ab,kw OR 'parkinson disease':ti,ab,kw | 14479 |
| #3 | Search #1 OR #2 | 14479 |
| #4 | MeSH descriptor: [Curcumin] explode all trees | 720 |
| #5 | ('Curcuma' OR 'Turmeric' OR 'Turmeric Yellow' OR 'Yellow, Turmeric' OR 'curcumine'):ti,ab,kw | 1206 |
| 6 | Search #4 OR #5 | 1745 |
| 6 | Search #3 AND #6 | 6 |
