# Supplementary material for: Pharmacological effects and mechanisms of curcumin in animal models of Parkinson’s disease: a systematic review and meta-analysis
Source: Front Pharmacol. 2026 Mar 9;17:1779921. doi: 10.3389/fphar.2026.1779921 (PMC13006684; doi:10.3389/fphar.2026.1779921)
Supplement: Supplementary file 2 [file Supplementaryfile3.docx]

Supplementary Material

# Supplementary Figures

## Supplementary Figure S1


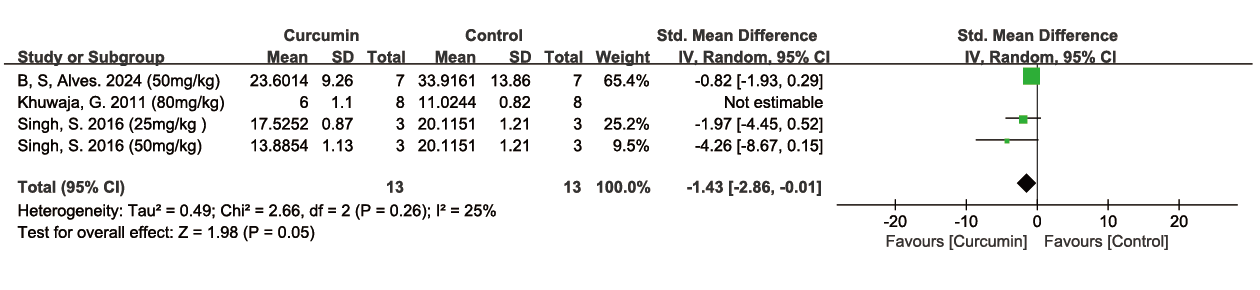


Supplementary Figure S1. Forest plot for the balance beam test after the removal of each study

## Supplementary Figure S2


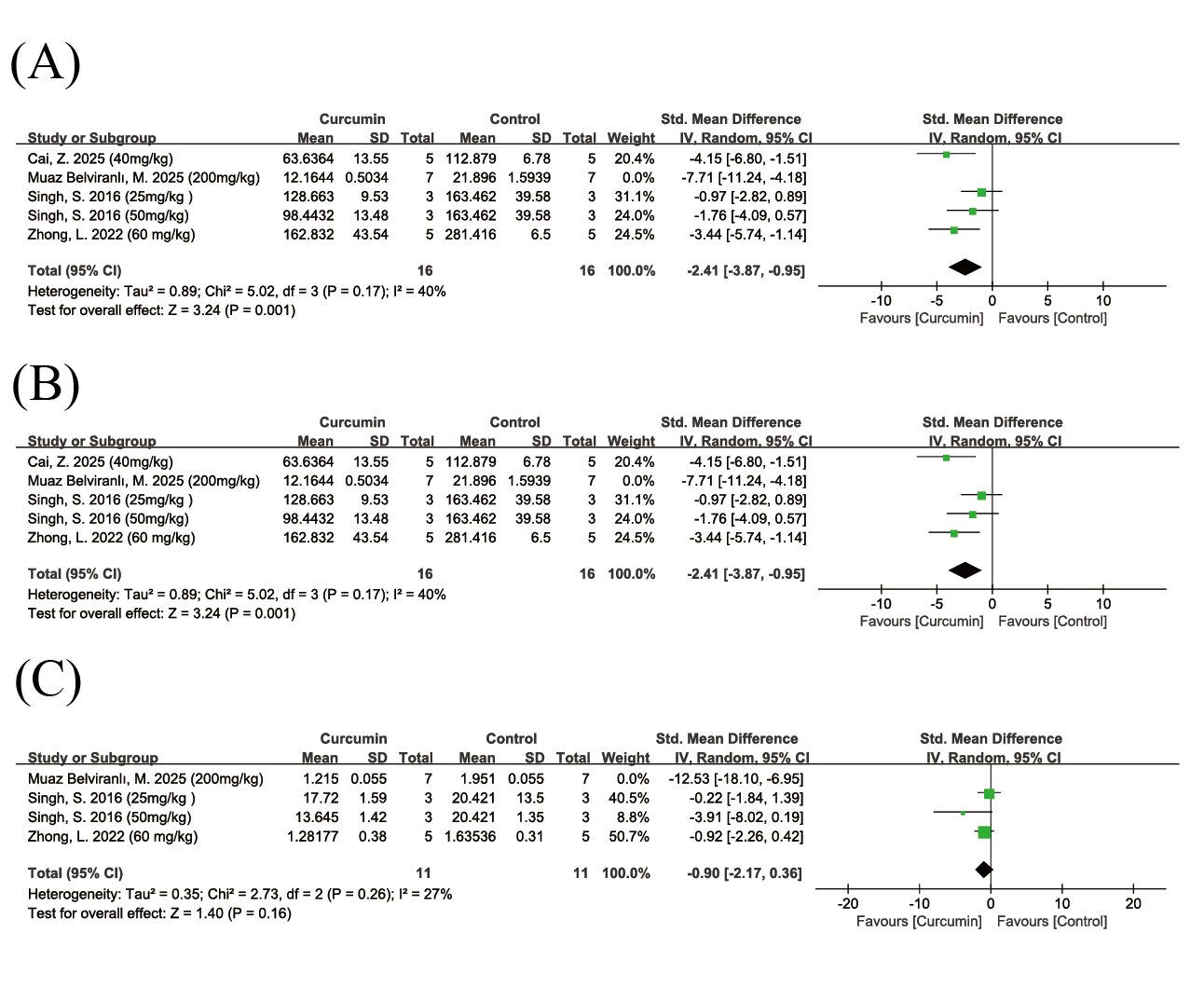


Supplementary Figure S2. Forest plots for IL-6, IL-1β, and TNF-α.

Note: Forest plot for IL-6 after the removal of each study (A); Forest plot for IL-1β after the removal of each study (B); Forest plot for TNF-α after the removal of each study (C).

## Supplementary Figure S3

##
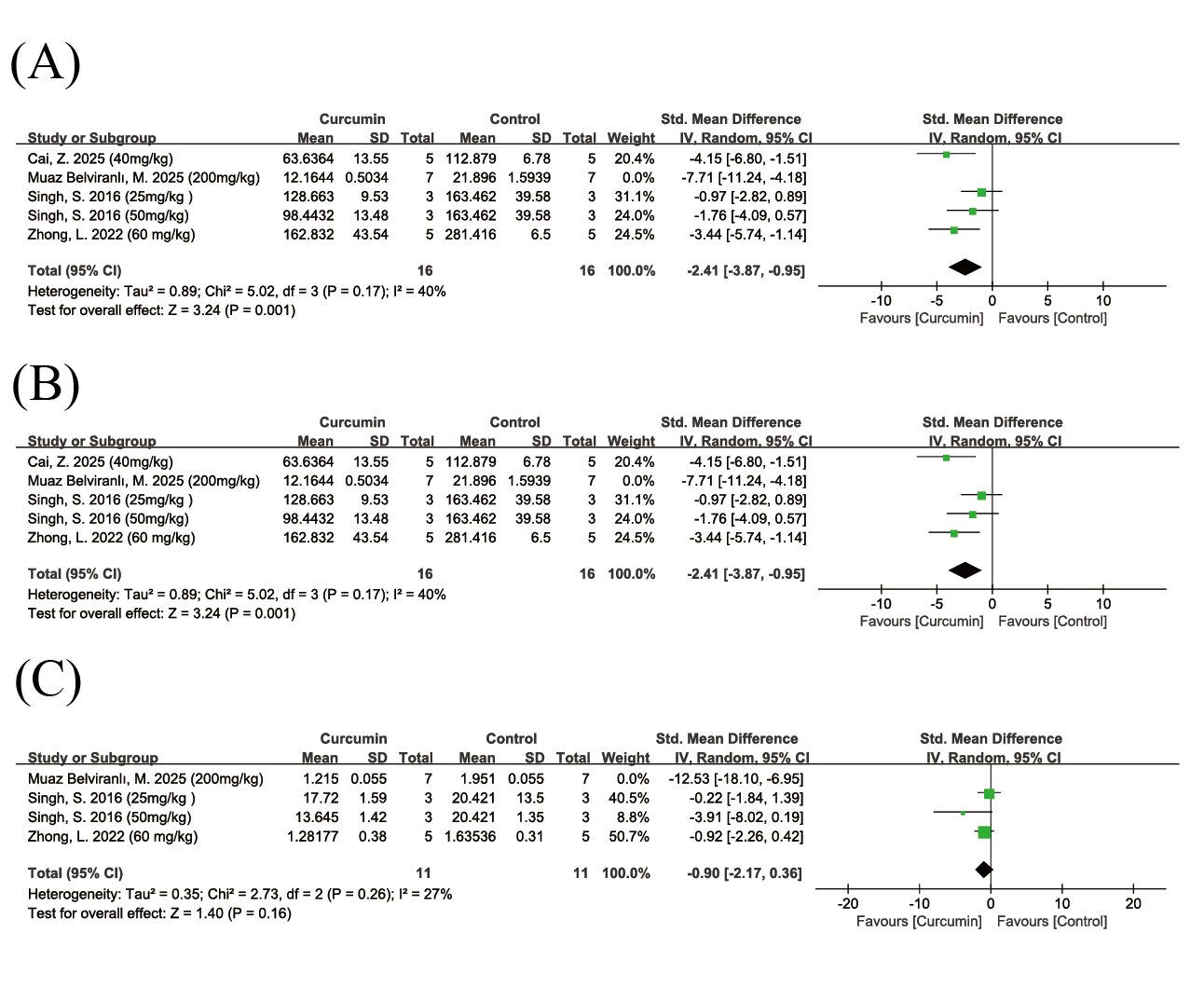


Supplementary Figure S3. Forest plots for SOD levels.

Note: Dose subgroup analysis of SOD levels (A); Species subgroup analysis of SOD levels (B); Forest plot of SOD following the exclusion of a single study that showed contrary results (C).

## Supplementary Figure S4


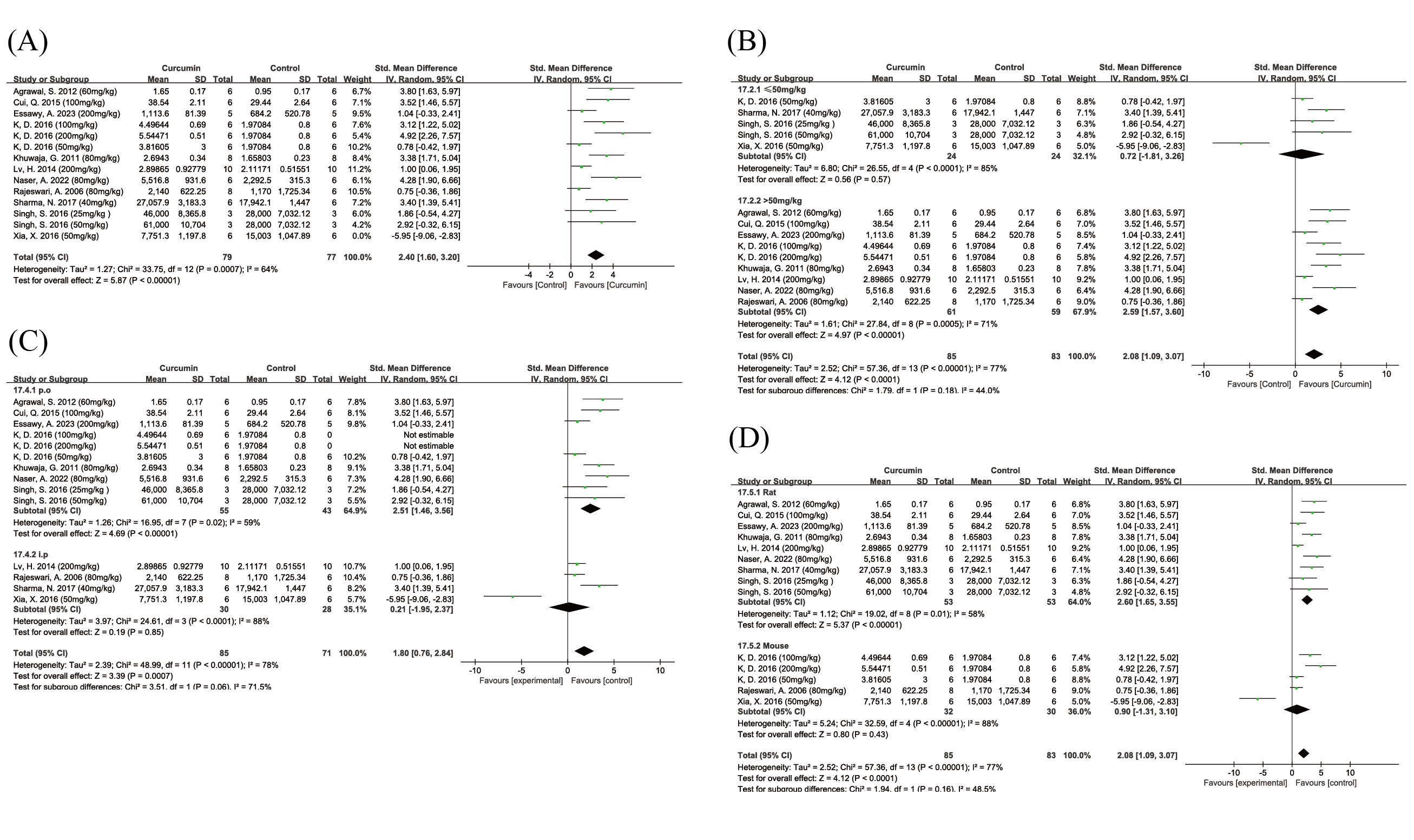


Supplementary Figure S4. Forest plot of GSH levels and subgroup analysis

Note: Forest plot of GSH following the exclusion of a single study that showed contrary results (A); Dose subgroup analysis of GSH levels (B); Subgroup analysis by route of administration for GSH levels (C); Species subgroup analysis of GSH levels (D).

## Supplementary Figure S5


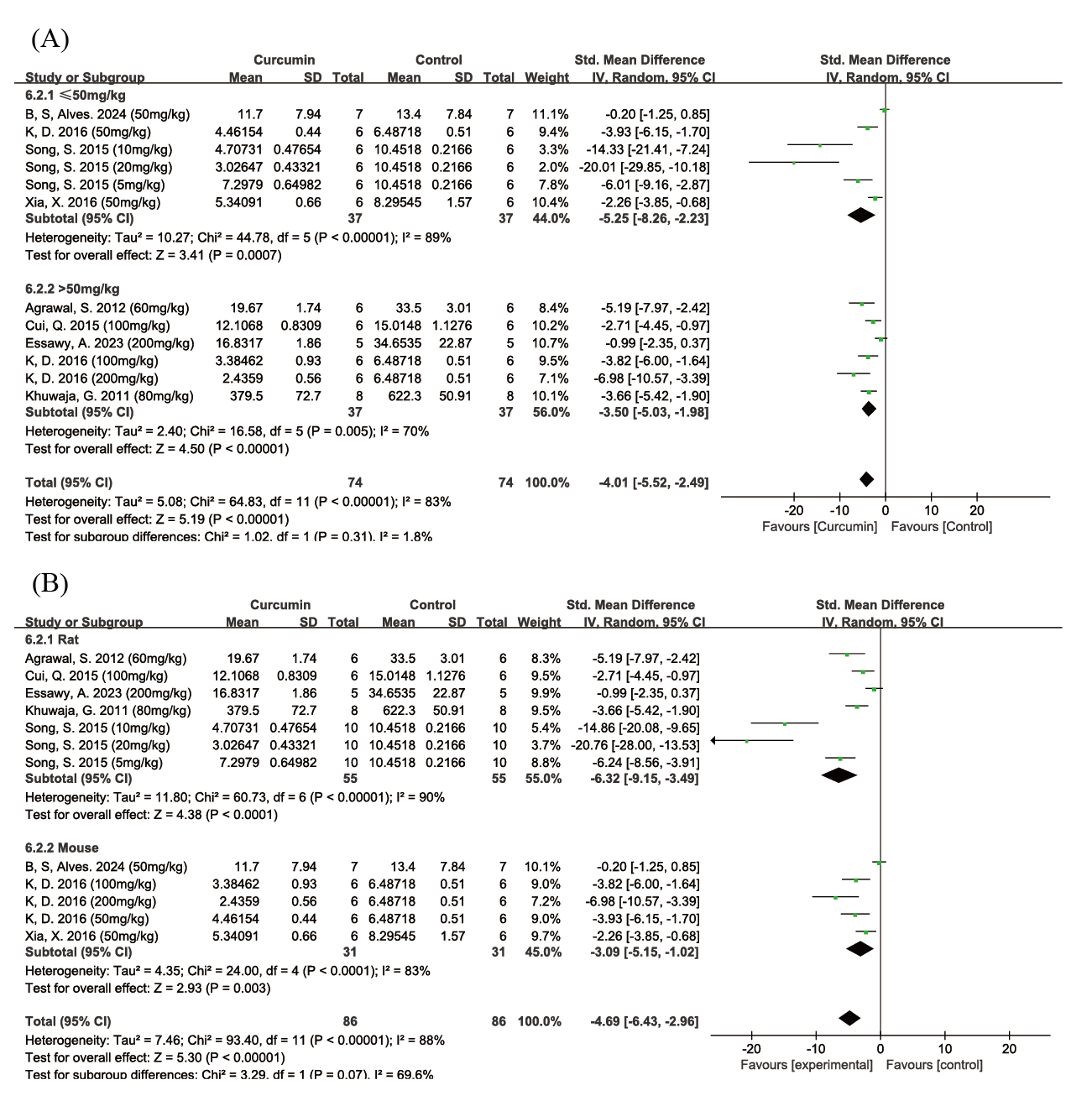


Supplementary Figure S5. Subgroup Analysis of MDA levels.

Note: Dose subgroup analysis of MDA levels (A); Species subgroup analysis of MDA levels (B).

## Supplementary Figure S6


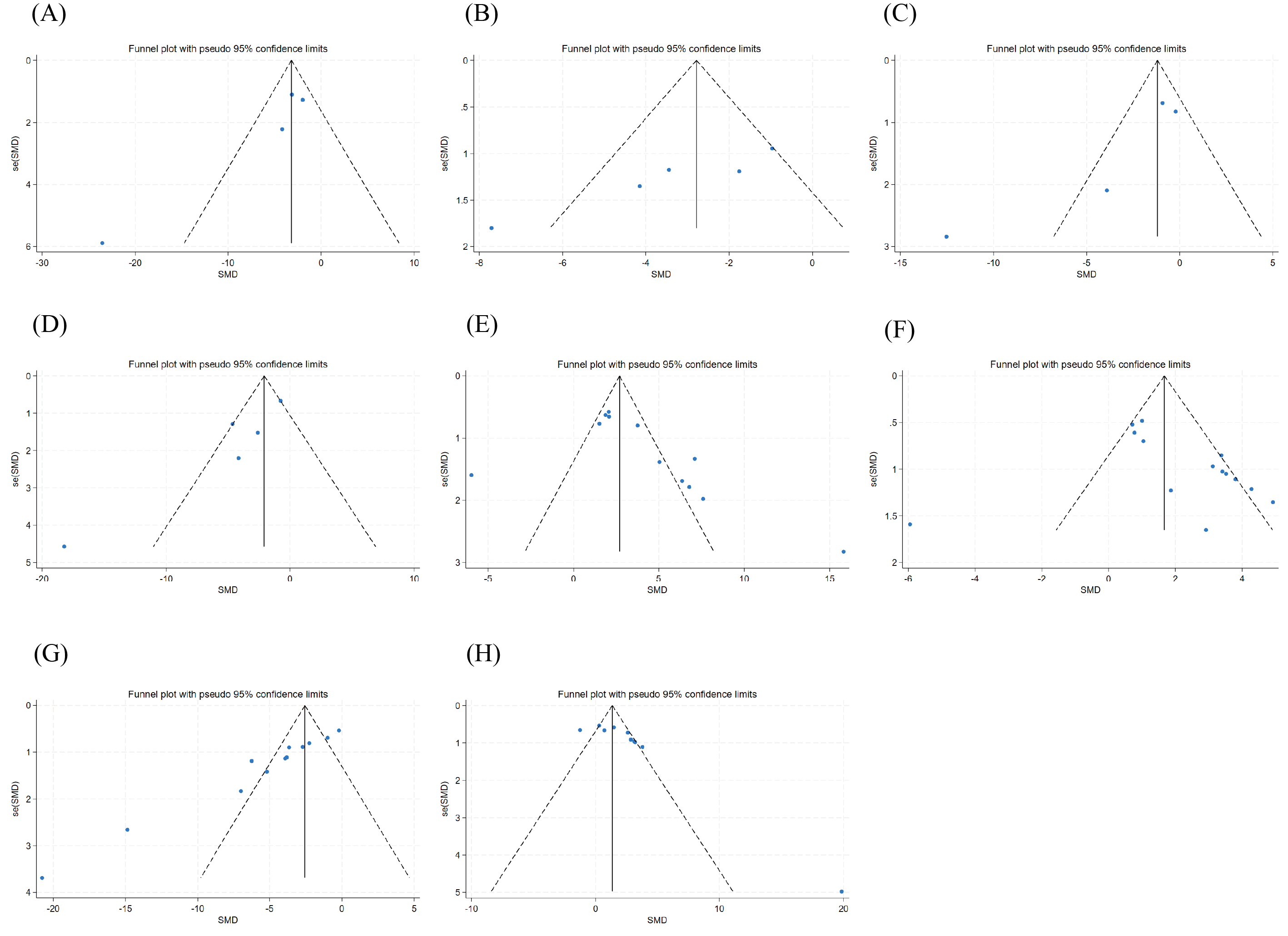


Supplementary Figure S6. Funnel plots of other outcome indicators.

Note: IL-6 (A); IL-1β (B); TNF-α (C); NO (D); SOD (E); GSH (F); MDA (G); CAT (H).

## Supplementary Figure S7


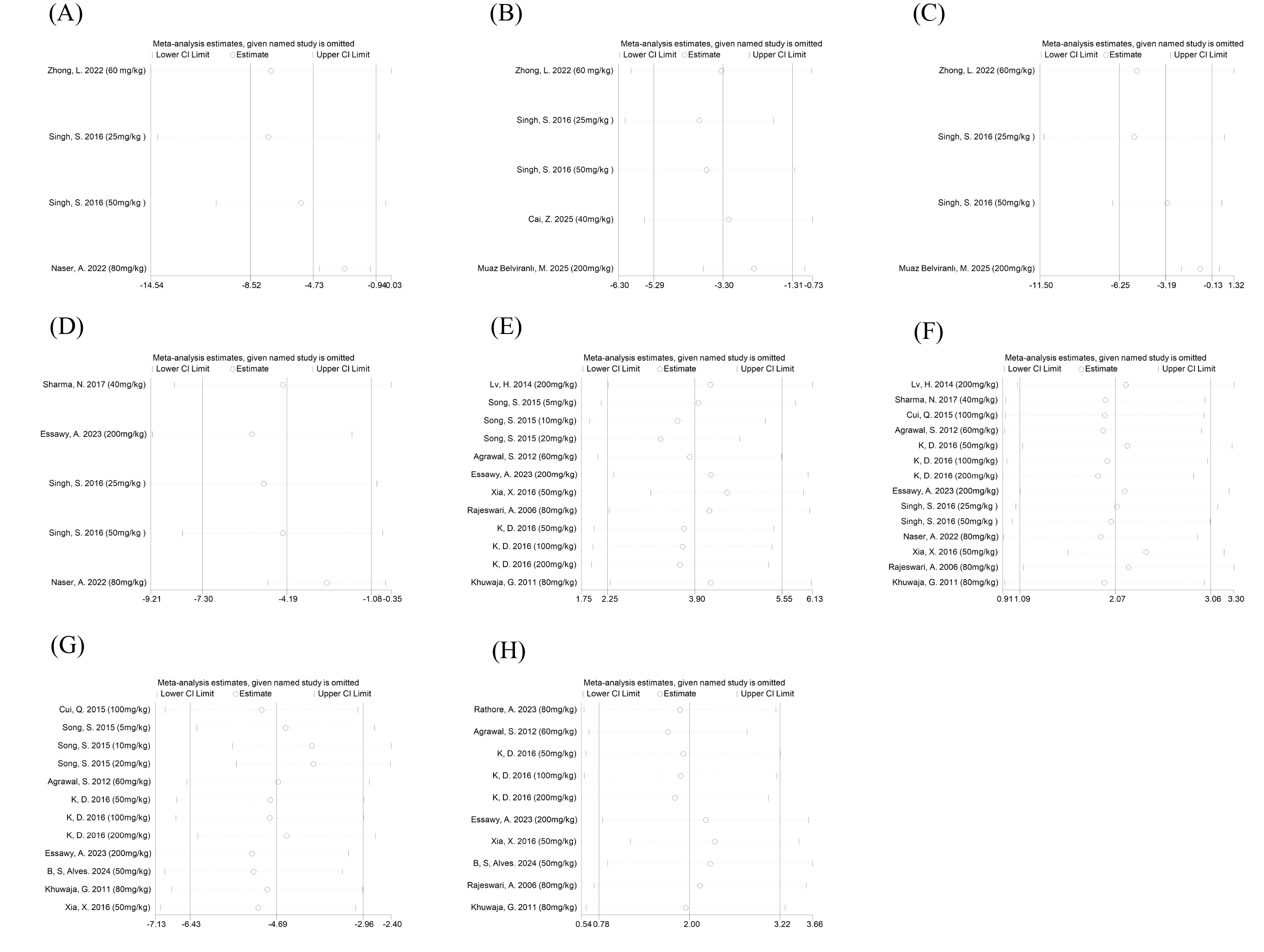


Supplementary Figure S7. Sensitivity analysis of other outcome indicators.

Note: IL-6 (A); IL-1β (B); TNF-α (C); NO (D); SOD (E); GSH (F); MDA (G); CAT (H).
