# Supplementary material for: Pharmacological effects and mechanisms of curcumin in animal models of Parkinson’s disease: a systematic review and meta-analysis
Source: Front Pharmacol. 2026 Mar 9;17:1779921. doi: 10.3389/fphar.2026.1779921 (PMC13006684; doi:10.3389/fphar.2026.1779921)
Supplement: Supplementary file 3 [file Supplementaryfile2.docx]

Supplementary Material

**Supplementary Table 1:** Egger's test of other outcome indicators.

| **Outcome** | **t** | **p** | **95% Conf. interval** | | **No. of studies (containing different doses)** |
| --- | --- | --- | --- | --- | --- |
| IL-6 | -2.92 | 0.100 | -8.981698 | 1.722801 | 4 |
| IL-1β | -5.43 | 0.012 | -12.42872 | -3.247842 | 5 |
| TNF-α | -2.80 | 0.107 | -10.44624 | 2.208164 | 4 |
| NO | -3.75 | 0.033 | -6.734896 | -0.5553 | 5 |
| SOD | 2.00 | 0.074 | -0.387535 | 7.042194 | 12 |
| GSH | 1.39 | 0.189 | -1.147573 | 5.214296 | 14 |
| MDA | -13.21 | 0.000 | -7.59075 | -5.399956 | 12 |
| CAT | 3.77 | 0.005 | 2.019886 | 8.401479 | 10 |

NOTE: Abbreviations: IL-6, interleukin-6; IL-1β, interleukin-1β; TNF-α, tumor necrosis factor-α; NO, Nitric Oxide; SOD, superoxide dismutase; GSH, Glutathione; MDA, malondialdehyde; CAT, Catalase
